# The population genetics of wild chimpanzees in Cameroon and Nigeria suggests a positive role for selection in the evolution of chimpanzee subspecies

**DOI:** 10.1186/s12862-014-0276-y

**Published:** 2015-01-21

**Authors:** Matthew W Mitchell, Sabrina Locatelli, Lora Ghobrial, Amy A Pokempner, Paul R Sesink Clee, Ekwoge E Abwe, Aaron Nicholas, Louis Nkembi, Nicola M Anthony, Bethan J Morgan, Roger Fotso, Martine Peeters, Beatrice H Hahn, Mary Katherine Gonder

**Affiliations:** Department of Biology, Drexel University, Philadelphia, Pennsylvania 19104 USA; Department of Biological Sciences, University at Albany - State University of New York, Albany, New York 12222 USA; Institut de Recherche pour le Développement (IRD) and Université Montpellier 1 (UM1), 34394 Montpellier, France; Forensic Biology Section, North Carolina State Crime Laboratory, Raleigh, North Carolina 27603 USA; Wildlife Conservation Society, Bronx, New York 10460 USA; Institue for Conservation Research, Zoological Society of San Diego, Escondido, CA 92027 USA; Ebo Forest Research Project, BP 3055, Messa Yaoundé, Cameroon; Wildlife Conservation Society - Tazania, Ruaha-Katavi Landscape, Iringa Tanzania; Environment and Rural Development Foundation, BP 189, Buea, Cameroon; Department of Biological Sciences, University of New Orleans, New Orleans, Louisiana 70148 USA; School of Natural Sciences, University of Stirling, Stirling , FK9 4LA, UK; Wildlife Conservation Society - Cameroon, BP 3055, Messa Yaoundé, Cameroon; Departments of Medicine and Microbiology, University of Pennsylvania, Philadelphia, Pennsylvania 19104 USA

## Abstract

**Background:**

Chimpanzees (*Pan troglodytes*) can be divided into four subspecies. Substantial phylogenetic evidence suggests that these subspecies can be grouped into two distinct lineages: a western African group that includes *P. t. verus* and *P. t. ellioti* and a central/eastern African group that includes *P. t. troglodytes* and *P. t. schweinfurthii*. The geographic division of these two lineages occurs in Cameroon, where the rages of *P. t. ellioti* and *P. t. troglodytes* appear to converge at the Sanaga River. Remarkably, few population genetic studies have included wild chimpanzees from this region.

**Results:**

We analyzed microsatellite genotypes of 187 wild, unrelated chimpanzees, and mitochondrial control region sequencing data from 604 chimpanzees. We found that chimpanzees in Cameroon and eastern Nigeria comprise at least two, and likely three populations. Both the mtDNA and microsatellite data suggest that there is a primary separation of *P. t. troglodytes* in southern Cameroon from *P. t. ellioti* north and west of the Sanaga River. These two populations split ~200-250 thousand years ago (kya), but have exchanged one migrant per generation since separating. In addition, *P. t. ellioti* consists of two populations that split from one another ~4 kya. One population is located in the rainforests of western Cameroon and eastern Nigeria, whereas the second population appears to be confined to a savannah-woodland mosaic in central Cameroon.

**Conclusions:**

Our findings suggest that there are as many as three genetically distinct populations of chimpanzees in Cameroon and eastern Nigeria. *P. t. troglodytes* in southern Cameroon comprises one population that is separated from two populations of *P. t. ellioti* in western and central Cameroon, respectively. *P. t. ellioti* and *P. t. troglodytes* appear to be characterized by a pattern of isolation-with-migration, and thus, we propose that neutral processes alone can not explain the differentiation of *P. t. ellioti* and *P. t. troglodytes*.

**Electronic supplementary material:**

The online version of this article (doi:10.1186/s12862-014-0276-y) contains supplementary material, which is available to authorized users.

## Background

**Figure 1:**
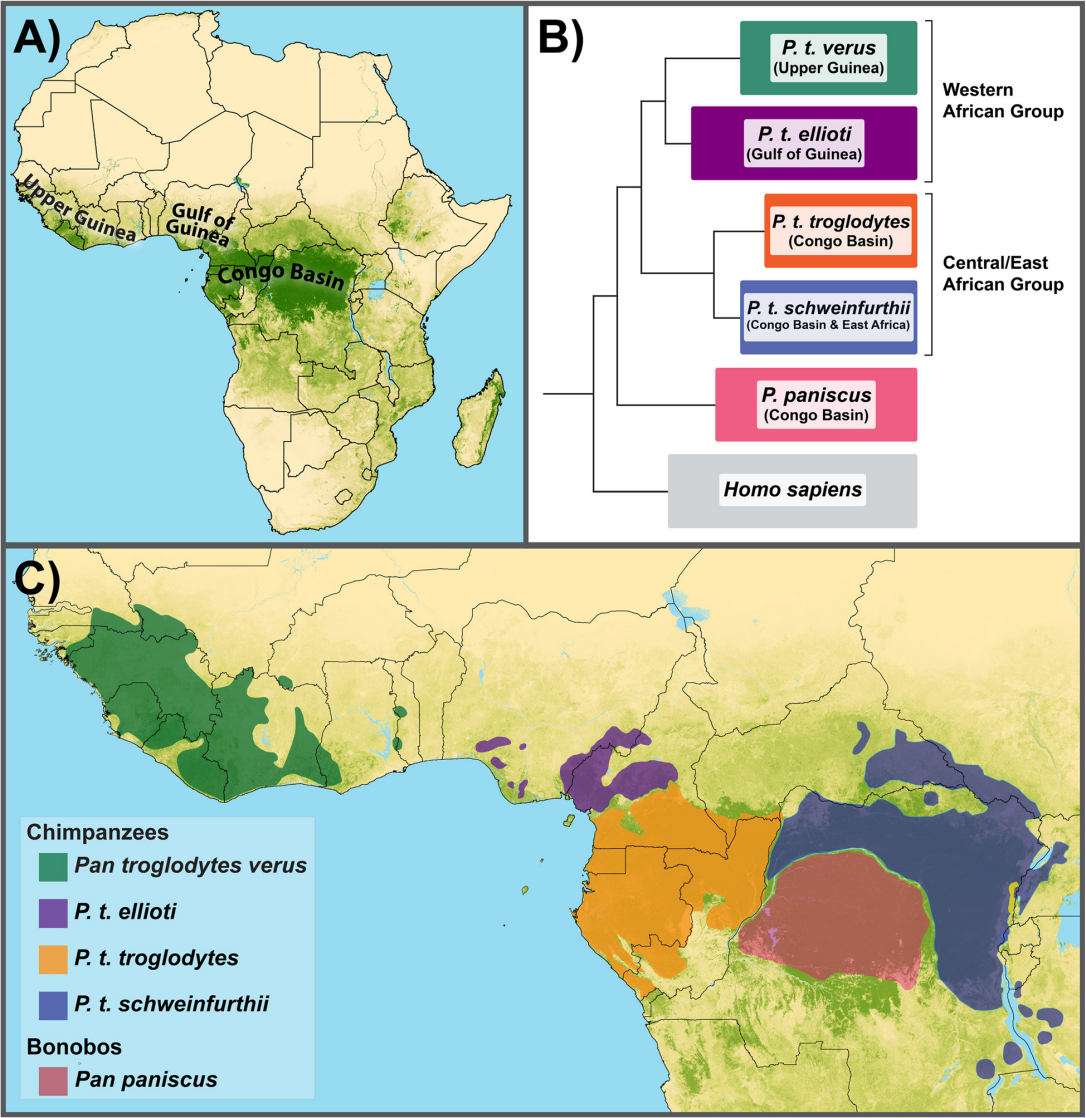
**Distribution and current phylogeny of** ***Pan.*** **(A)** Major rainforest biomes of tropical Africa. **(B)** Phylogeny of chimpanzees, bonobos and humans using whole genome data, adapted from Prado-Martinez *et al*. [8]. **(C)** Distribution of chimpanzee subspecies and bonobos across tropical Africa.

Chimpanzees (*Pan troglodytes*) are still widely distributed across sub-Saharan Africa, and the species exploits a wide range of habitats including rainforests, ecotones and savannas [1-3]. Across this range they exhibit considerable genetic [4], behavioral [5] and ecological diversity [3]. Studies consisting mostly of samples from wild-born captive chimpanzees have given great insights regarding the phylogenetic history of this species, but genetic data from wild individuals remain sparse. Recent studies of captive wild-born chimpanzees include datasets such as complete mitochondrial (mt) genomes [6], genome-wide single nucleotide polymorphisms (SNPs) [7], and complete genome sequences [8]. The overall picture to emerge from these studies suggests that chimpanzees are divided into two geographically- and genetically-defined groups: a western African group that includes *P. t. verus* and *P. t. ellioti* and a central/eastern African group that includes *P. t. troglodytes* and *P. t. schweinfurthii* [8] (Figure 1). This classification is consistent with earlier studies using mtDNA control region sequence diversity from wild individuals from Nigeria and adjacent parts of Cameroon, which suggested that animals in this region form a genetically distinct population of chimpanzees [9,10], now widely recognized as *P. t. ellioti* [11]. These studies have also been important for understanding differences in subspecies population histories, and how these histories are connected to landscape and forest history. For example, the western and central/eastern groups appear to have split from one another very early in the history of this species. Analysis of complete genomes suggests that since their genetic and geographic separation, these groups have experienced markedly different demographic histories, and the subspecies within each group show different patterns of population growth and decline throughout their respective histories [8].

While the phylogenetic relationships among chimpanzee subspecies are now well-resolved, their population structure, migration patterns and patterns of population growth still remain unknown, particularly in areas where the ranges of subspecies converge. This is mostly due to a lack of fine-scale geographic sampling of wild chimpanzee populations around areas where subspecies overlap, which has revealed mechanisms of diversification in gorillas [12]. Analysis of mtDNA from wild chimpanzees from Nigeria and Cameroon has suggested that the Sanaga River delimits *P. t. ellioti* from *P. t. troglodytes* in southern Cameroon [9]. This split is old, but incomplete, as there is a zone in central Cameroon where the ranges of the mtDNA haplotypes that define these two subspecies overlap. These findings suggest that some introgression may have occurred between these two subspecies [4,10,13] or that a pattern of isolation-by-distance (IBD) might be revealed by more fine scale population sampling across this region.

The Sanaga River has been proposed to delimit the distributions of several primate species and subspecies [12,14-18] including chimpanzees [4,19]. This region of Africa also contains diverse habitats. Cameroon lies at the intersection of two major rainforest biomes, the Guinean and Congolian rainforests [20] (Figure 1a). These two forests converge in central Cameroon and are connected by a zone of open woodland, savannah and riparian forest [20,21] which has been termed an ‘ecotone’ [22]. Therefore, denser geographic sampling and more comprehensive genetic data are necessary to tease apart the relative contributions of forest history and biogeographic boundaries in driving patterns of genetic variation in chimpanzees.

**Figure 2:**
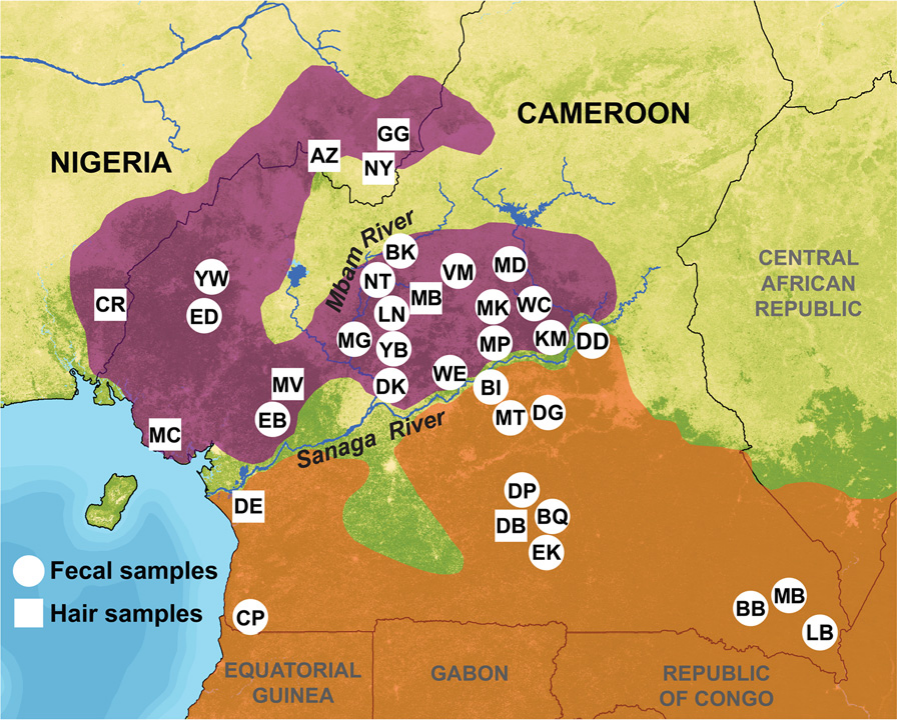
**Sample locations of chimpanzees.** Locations spanned Cameroon and eastern Nigeria. Probable distributions of *P. t. ellioti* (purple) and *P. t. troglodytes* (orange) ranges are shown. Circles denote both hair and fecal samples were collected at the location. Squares denote only hair samples were collected.

This study uses DNA extracted from fecal and hair samples to examine the population structure and genetic history of chimpanzees from Cameroon and eastern Nigeria at a fine geographic scale across this ecologically diverse region (Figure 2). The overall goals of the study were to use mtDNA haplotypes along with microsatellite genotype profiles of wild individuals to: (1) test between a variety of hypotheses for the presence and type of population structure (Table 1), including (*i*) panmixia, (*ii*) isolation-by-distance, (*iii*) population structure with complete isolation, and (*iv*) population structure with ongoing migration; (2) test the specific hypothesis that the Sanaga River has been important in delimiting the range of *P. t. ellioti* from neighboring *P. t. troglodytes*; and (3) compare the demographic histories of these two subspecies by inferring the time to their most recent common ancestor (T_MRCA_), their historical and current effective population sizes, and finally, the rates and directions of migration between them.

**Table 1:** **Hypotheses and predictions**

**Models of population history**	**Patterns of genetic differentiation**		**Demographic history**
	**mtDNA**	**Microsatellites**	
**Panmixia**	One cluster of haplotypes spanning entire study region; Majority of genetic variation found between individuals	No evidence of discernable population structure into *k* > 2 demes	All individuals equally divergent from one another
**Isolation**-**by**-**distance**	Positive linear relationship between genetic and geographic distance	Positive linear relationship between genetic and geographic distance	Populations closer to one another share a more recent relationship than those more distant for one another
**Population structure with complete isolation**	Haplotypes cluster into discrete and geographically distinct groups	Individuals cluster into discrete, geographically distinct groups	No evidence of recent migration between populations, but ancestral polymorphism may still be present
**Population structure with ongoing migration**	Haplotypes cluster into geographically distinct groups with overlap	Individuals cluster into geographically distinct groups with some individuals showing evidence of admixture	Evidence of recent migration between populations that is distinguishable from ancestral polymorphism

## Results and discussion

### Dataset preparation

We calculated pairwise estimates of relatedness for all pairs of genotyped individuals using the software program COANCESTRY [23] and removed duplicate individuals and those that were closely related to one another. A total of three individuals with a relatedness index value [24] above 0.75 were determined to be either identical individuals or highly related and were excluded from all further analyses. The analyses presented here include mtDNA sequencing data from 604 sequences from 35 sampling locations, and microsatellite data were included from 187 unrealated individuals from 28 sampling locations (Additional file 1).

In order to ensure that all of the genetic markers were selectively neutral, and therefore suitable for further analysis, the data were subjected to various neutrality tests. We found that all microsatellite loci met expectations of Hardy-Weinberg equilibrium and expectations of neutrality (Additional files 2 and 3). We further subjected the microsatellite loci to an outlier test based on observed heterozygosity and *F*_*ST*_, and all 21 loci fell within the acceptable range of neutrality (Additional file 4).

### mtDNA diversity analysis

**Figure 3:**
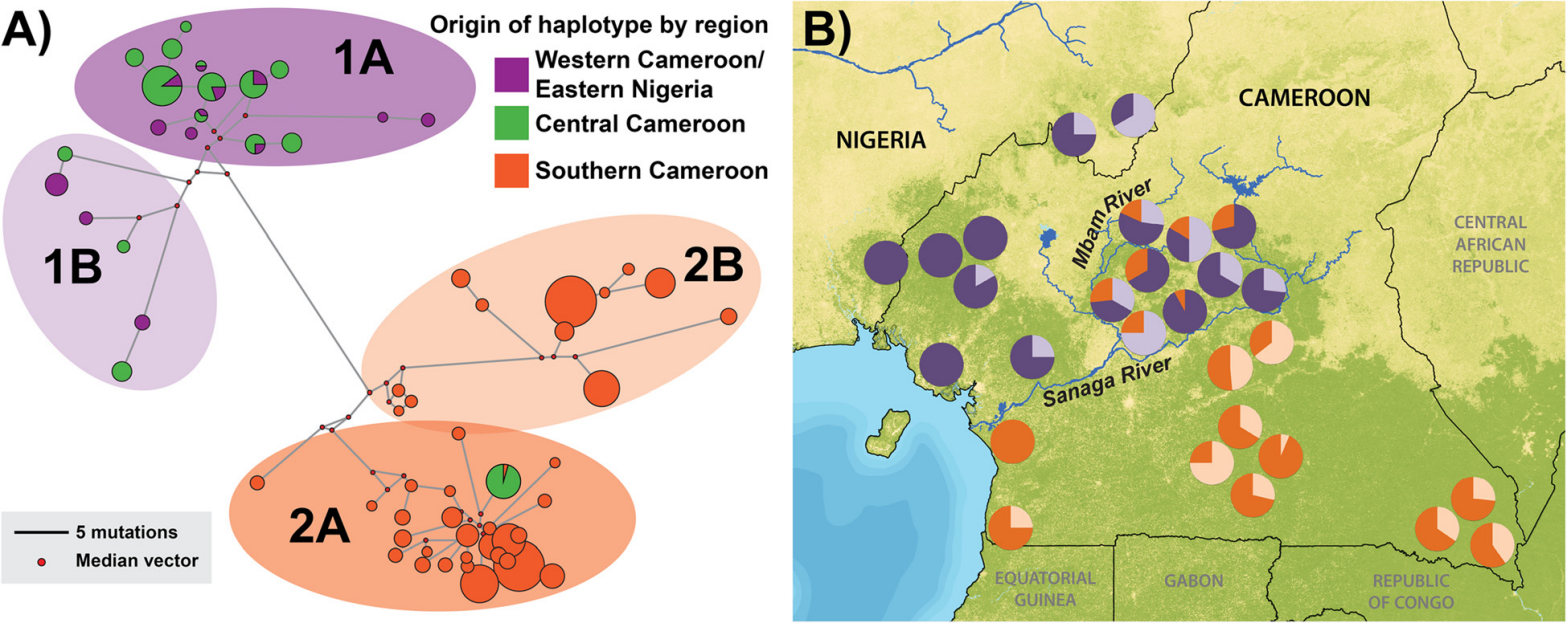
**mtDNA haplotype network and map.** **(A)** Median-joining haplotype network of mtDNA HVRI locus generated using Network 4.5. Each cluster is color coded to display the inferred geographic origin of individuals; (*i*) purple representing western Cameroon and eastern Nigeria (north of the Sanaga River and west of the Mbam River), (*ii*) green representing central Cameroon (north of the Sanaga and east of the Mbam), and (*iii*) orange representing southern Cameroon (south of the Sanaga). Haplotypes cluster into two main groups (1 and 2), and 4 sub-groups (1A, 1B, 2A and 2B). **(B)** Pie charts show the frequency distribution of mtDNA haplogroups (1A, 1B, 2A and 2B) across the study area.

We constructed a median-joining mtDNA haplotype network (Figure 3a) and plotted a frequency distribution of inferred haplotypes found at each sampling location across Cameroon and Nigeria (Figure 3b). The results were similar to observations from previous studies [10,13]. Specifically, there are two primary mtDNA haplogroups, each comprised of two distinct haplotypes, found across the study area. One haplogroup (Figure 3a light purple and dark purple) occurs only in individuals north of the Sanaga River (range of *P. t. ellioti*). The second haplogroup (Figure 3a light orange and dark orange) occurs in *P. t. troglodytes* primarily south of the Sanaga. However, these two haplogroups overlap with each other in the ecotone in central Cameroon, north of the Sanaga River and east of the Mbam River. Interestingly, the *P. t. troglodytes*–like mtDNA sequences found in the ecotone belong to a single sub-type within haplogroup 2A. This suggests either a pattern of historic gene flow between the subspecies, which is consistent with other studies [10,13], or alternatively that there was a single migration event from south to north of the Sanaga followed by the proliferation of this haplotype exclusively within central Cameroon.

Overall, the results of the mtDNA haplotype analysis suggest that panmixia does not describe the population structure of chimpanzees. There is clear evidence that there are two main mtDNA haplogroups: a group in eastern Nigeria and western Cameroon and a second group in southern Cameroon. The geographic distributions of these groups converge with one another in central Cameroon, with an abrupt transition between them along the Sanaga River. Overlap between these groups appears to be confined to the ecotone of central Cameroon, which also coincides with the confluence of the Sanaga with the Mbam River--another river of biogeographic significance for primates [16]. It is also worthy to note that *P. t. ellioti* in central Cameroon are more diverse than those in western Cameroon/eastern Nigeria (Figure 3b). Lower haplotype diversity in western Cameroon and eastern Nigeria, which has been proposed to be an area of putative Pleistocene refugia [25,26], may either be the result of local extinctions of haplotypes, or a diversification of mtDNA haplotypes as the result of increased habitat variation in central Cameroon.

We grouped results of the Analysis of Molecular Variance (AMOVA) (Table 2) for mtDNA sequences according to variation: (*i*) within populations (Φ_ST_); (*ii*) among populations in groups (Φ_SC_); and (*iii*) among groups north or south of the Sanaga River (Φ_CT_), the major population partition indicated in previous studies [9,10]. Dividing the haplotypes by their origins north versus south of the Sanaga River accounted for 52.41% of the variation among groups, whereas 43.22% of the variation occurred within sampled populations within these groups. This population pattern is markedly different from the partitioning of genetic variation found in *P. t. schweinfurthii* [27] where less than 2% of the genetic variation were reported to have accounted for the differences between *P. t. schweinfurthii* groups that were separated from one another by more than a 600 km straight-line distance (Table 2), whereas the groups included in this AMOVA are separated by no more than 30 km at their points of closest sampling north versus south of the Sanaga River (Figure 2). Similarly, results for a spatial AMOVA (SAMOVA) [28] that assumed two populations (*k* = 2) were present across the study area confirmed the geographic grouping of sampling locations north and south of the Sanaga River. The division of haplotypes according to geographically homogeneous groups, accounted for 49.37%, and 45.56% of the variation within sample locations within these groups (Additional file 5). This pattern was statistically significant and recapitulated the results of the AMOVA (Table 2).

**Table 2:** **Analsis of Molecular Variance** (**AMOVA**) **for mtDNA HVRI**

**Partition**	**Fixation indices**	**Variance components**	**Percentage of variation**	**Significance**
	North (*P. t. ellioti*) versus south of Sanaga (*P. t. troglodytes*)^a^			
Among groups (Φ_CT_)	00.52	10.09	52.41	*p* < 0.05
Among populations in groups (Φ_SC_)	00.56	00.84	04.37	*p* < 0.05
Within populations (Φ_ST_)	00.09	08.32	43.22	*p* < 0.05
	DRC versus eastern forests (both *P. t. schweinfurthii*)^b^			
Among groups (Φ_CT_)	00.00	00.00	00.00	Not significant
Among populations in groups (Φ_SC_)	00.10	00.05	10.23	*p* < 0.05
Within populations (Φ_ST_)	00.10	00.45	89.77	*p* < 0.05

^a^AMOVA results of data from this study.

^b^AMOVA analysis reported by Goldberg and Ruvolo [27]. The samples included eastern chimpanzees (*P. t. schweinfurthii*) divided into two groups of samples recovered from chimpanzees in northeastern Democratic Republic of Congo (DRC) and ‘eastern forests’ that includes sampling locations in Rwanda, Tanzania and Uganda. These two groupings are separated from one another by more than 600km straight-line distance.

We generated two sets of mismatch distributions to examine whether the sampled populations had experienced recent botttlenecks, population expansions or demographic stability (Additional files 6 and 7). Individuals were grouped into either two or three separate groups, as identified by microsatellite cluster analysis. None of the mismatch distributions were found to be significantly different from the null model, which indicates demographic stability of all populations included in the analyses (Additional files 6 and 7) [29].

### Microsatellite genotype analysis

**Figure 4:**
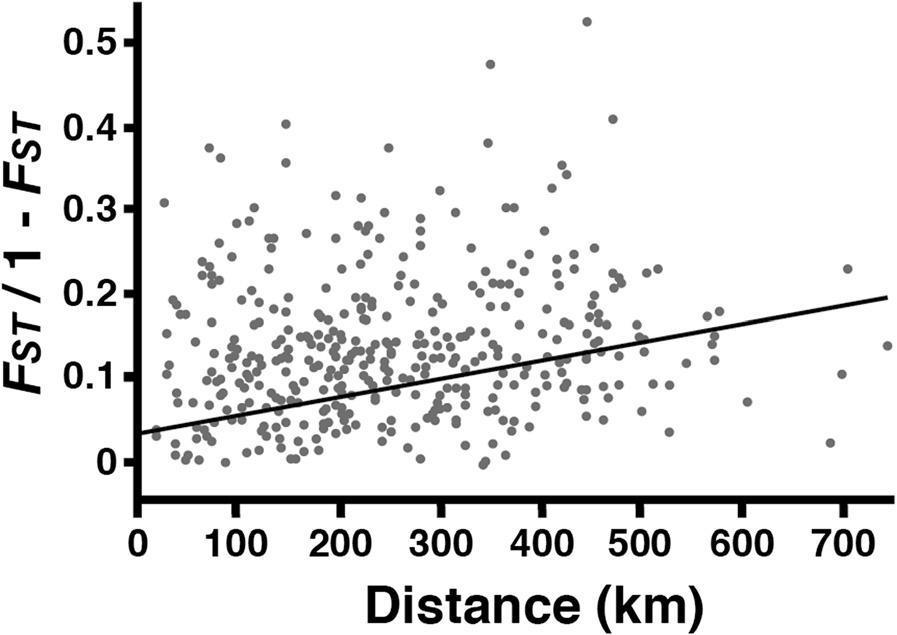
**Isolation**-**by**-**distance.** Results of a Mantel test performed to identify a correlation between genetic differentiation and the geographic distance between sampling locations (Figure 2). Points on the chart represent pairwise comparisons and the trend line represents linear correlation (p = 0.0045, R^2^ = 0.0186).

Mantel tests of 21 microsatellite loci (Figure 4) revealed a significant (p < 0.05) pattern of isolation-by-distance across the study area as a whole. However, the relationship between genetic and geograpahic distance is weak. The R^2^ value is low (R^2^ = 0.0186) and overall accounts for for less than 2% of the total genetic variation present across the study area. Because isolation-by-distance only weakly explains the population structure of chimpanzees, we next examined the number and distribution of populations across the study area.

**Figure 5:**
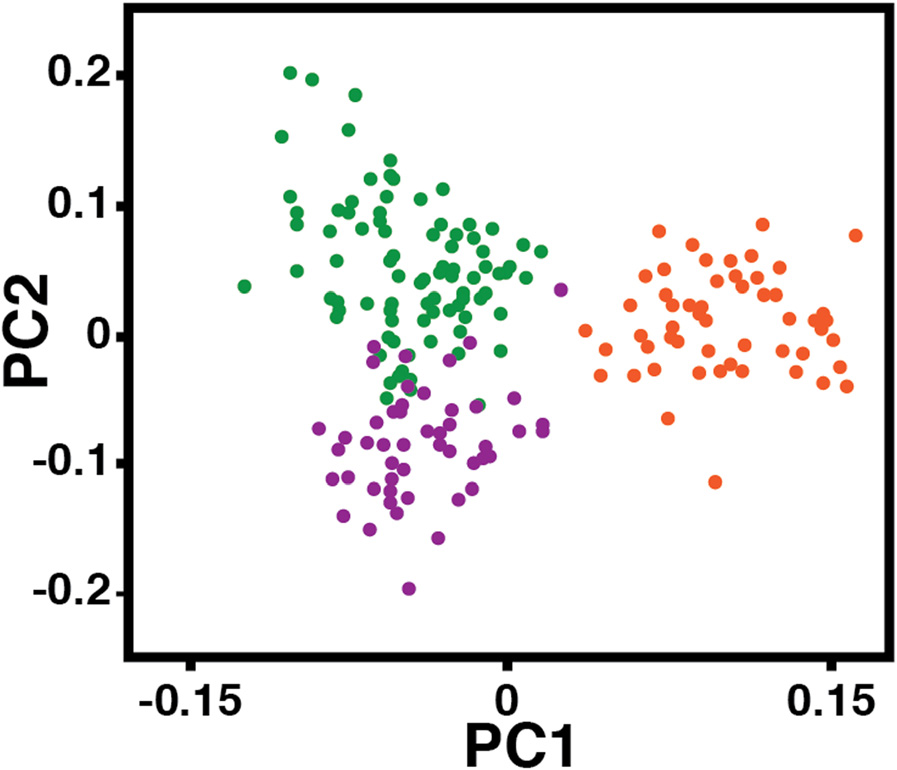
**Principal Components Analysis** **(PCA).** PCA generated on the basis of individual genotypes. Individuals are color coded according to geographic origin; (*i*) purple – western Cameroon and eastern Nigeria, (*ii*) green – central Cameroon, and (*iii*) orange – southern Cameroon. PCs 1 and 2 (shown) represent the eigenvectors that accounted for 71 and 21%, respectively, of the total extracted variation.

A Principal Components Analysis (PCA) was carried out to examine how many different gene pools were present across the study region. The PCA shown in Figure 5 classified the first four eigenvectors as significant (p < 0.05). The first Principal Component (PC 1) separated chimpanzees north and south of the Sanaga River (*P. t. ellioti* and *P. t. troglodytes*), and accounted for 71% of the total extracted variation in the dataset. PC 2 accounted for 21% of the total extracted variation and separated *P. t. ellioti* chimpanzees into two groups: one cluster of individuals mostly from the forests of western Cameron (further designated as: *P. t. ellioti* (Rainforest), purple) and another cluster of individuals from the ecotone in central Cameroon (further designated as: *P. t. ellioti* (Ecotone), green). PC 3 and PC4 accounted for 5% and 3% respectively, and separated small clusters of individuals from the same sampling locations from the rest of the dataset. These final PCs were sample location specific and are possibly the result of sampling bias (i.e. sampling of related individuals below our relatedness threshold).

**Figure 6:**
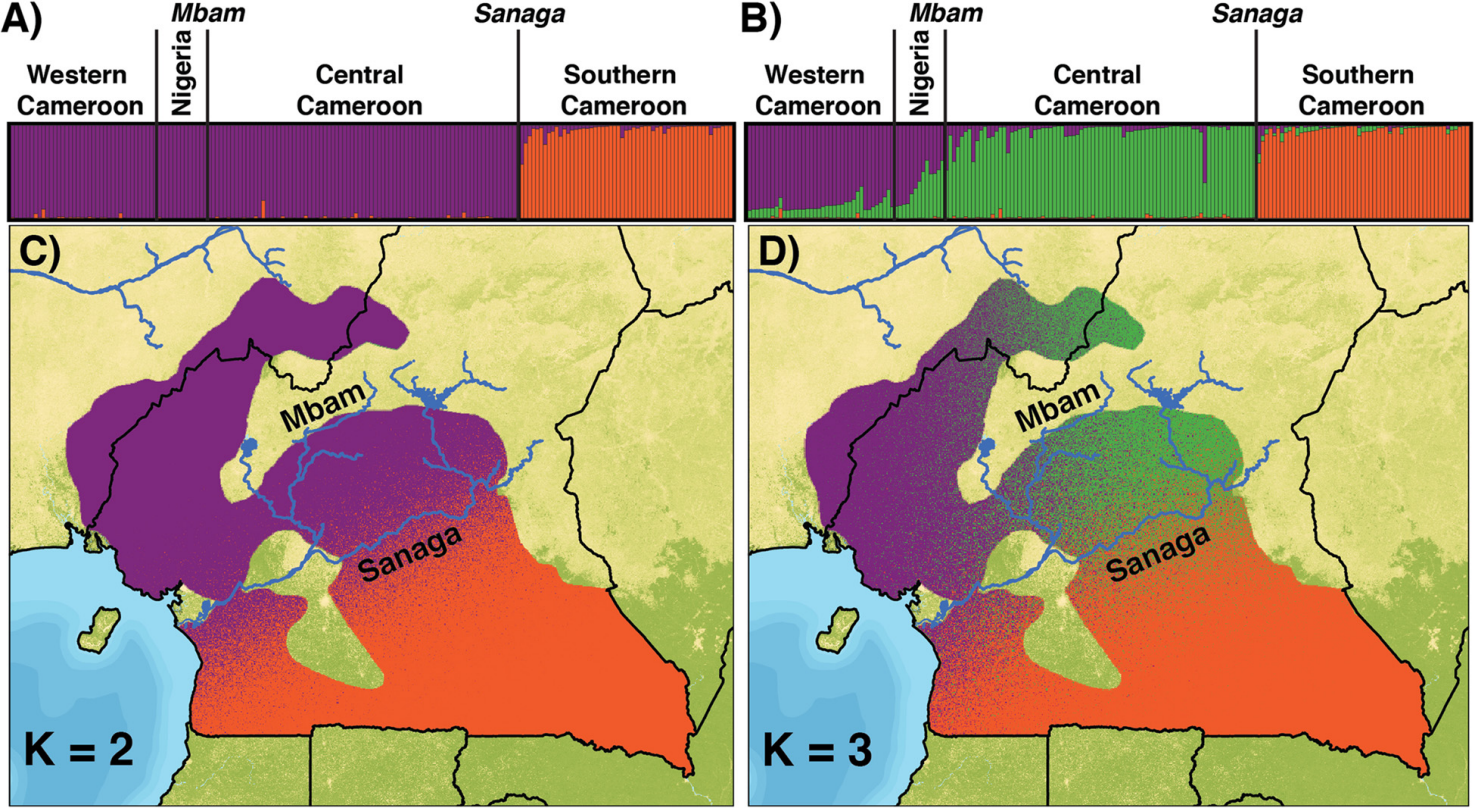
**Cluster analysis and spatial interpolations.** **(A and B)** TESS bar plots for *K* = 2-3. Each vertical line represents an individual, and colors represent their inferred ancestry from *K* ancestral populations. Individuals are ordered by their geographic sampling location. **(C and D)** Spatial interpolations of the *Q* matrices generated by the program TESS Ad-Mixer for *K* = 2-3. Spatial interpolations were plotted on probable extents of chimpanzee ranges in Nigeria and Cameroon.

Results of the cluster analysis in TESS [30] are shown in Figures 6a and b. We used several methods to infer the maximum number of populations, *K*, represented by this dataset. Deviance Information Criteria (DIC) [31] values suggest that *K*_*MAX*_ is 2 (Additional file 8a). In contrast, the *post hoc* statistic, ΔK [32], suggests a *K*_*MAX*_ of 3 (Additional file 8b). Overall, these results suggest that there is a primary separation along the banks of the Sanaga River. *P. t. ellioti*, (purple) is found north and west of the river, whereas *P. t. troglodytes* (orange) occurs south of the Sanaga (Figure 6a). There is additional evidence of a population subdivision within *P. t. ellioti*: a population located in the forests of western Cameron (*P. t. ellioti* (Rainforest), purple) and another population located in the ecotone in central Cameroon (*P. t. ellioti* (Ecotone), green) (Figure 6b).

The spatial interpolations generated by TESS Ad-Mixer [33] show the separation of these two populations (*P. t. ellioti*, purple and *P. t. troglodytes*, orange) corresponds to banks of the Sanaga River (Figure 6c). Setting *k* = 3, which was supported by the ΔK statistic and the PCA, provided evidence of further subdivision within *P. t. ellioti*. Setting *k* = 2 and *k* = 3 using TESS (Figure 6) distinguished the same populations as PC1 and PC2 (Figure 5). The TESS Ad-Mixer [33] results show that this subdivision exists exclusively north of the Sanaga River, and the east–west division of populations (purple and green) coincides with the Mbam River, although this division is much less pronounced than the division of *P. t. ellioti* and *P. t. troglodytes* at the Sanaga River (Figure 6d), and there is predicted overlap between these two groups in Nigeria, north of the Mbam.

Values for three measures of genetic differentiation, *D*^2^ [34], *R*_*ST*_ [35], and *δμ*^*2*^ [36], are shown in Additional file 9 between the three populations identified in the cluster analyses (Figures 5 and 6). All three measures were correlated with one another; *D*^2^ and *R*_*ST*_, *r*^2^ = 0.94; *D*^2^ and *δμ*^*2*^, *r*^2^ = 0.58; and *R*_*ST*_ and *δμ*^*2*^, *r*^2^ = 0.81; and all pairwise differences were significantly different from null expectations of the data (Additional file 9). Genetic differentiation was highest between populations of chimpanzee across the Sanaga River (*P. t. ellioti* versus *P. t. troglodytes*) and lowest between populations of chimpanzee north of the Sanaga River (*P. t. ellioti* (Rainforest) versus *P. t. ellioti* (Ecotone)). Additional file 9 shows the estimated number of migrants exchanged between populations per generation (2N*m*) as calculated using in the Arlequin version 3.5 software package [37]. Between 1 and 2 migrants per generation are exchanged between populations located north vs. south of the Sanaga River, and approximately 11 migrants exchanged between the two populations found north of the Sanaga River in western and central Cameroon, respectively.

Allele richness and number of private alleles by population, corrected for unequal population size, is shown in Additional files 10a and 10b. Allele richness varied considerably between populations, with *P. t. troglodytes* displaying almost twice as many distinct alleles per locus compared to *P. t. ellioti* (Rainforest) and *P. t. ellioti* (Ecotone). However, the mean number of private alleles per locus did not vary considerably between regions, although we observed a similar pattern, with *P. t. troglodytes* exhibiting the greatest number of private alleles, when corrected for unequal sample size. Additional file 10c shows the mean number of shared private alleles between the three combinations of population pairs, when corrected for equal population size. Interestingly, the highest number of shared private alleles occurs between *P. t. ellioti* (Rainforest) and *P. t. troglodytes*, with the other two population pairs exhibiting similar numbers of shared alleles.

### Population history

We pooled the mtDNA and microsatellite data together for analysis in IMa [38], which allowed for the estimation of several population parameters (Table 3), including descendant (N_1_ and N_2_) and ancestral (N_A_) population sizes, time of divergence and migration rates between each of the three populations recovered by the cluster analysis (Figure 6) and PCA (Figure 5). All three population comparisons yielded well-resolved posterior probability distributions for each population parameter (Additional files 11, 12 and 13). Both *P. t. ellioti* (Rainforest) and *P. t. ellioti* (Ecotone) last shared a common ancestor with *P. t. troglodytes* approximately 200 to 250 kya, which is consistent with other studies using similar data [39]. The two *P. t. ellioti* populations last shared a common ancestor with each other much more recently, about 4 kya. The results of these analyses also included estimates of ancestral and descendant effective population sizes for all pairs of populations. Estimates of effective population size for the present *P. t. ellioti* (Rainforest) population were consistent across runs, showing an effective population size of approximately 2,000 individuals. There was considerably more variation detected between comparisons for effective population sizes of *P. t. ellioti* (Ecotone) and *P. t. troglodytes*. In *P. t. ellioti* (Ecotone), effective population size was estimated to be between approximately 1,000 and 2,500 individuals. The current effective population size of *P. t. troglodytes* in southern Cameroon, may range from 3,000 to 10,000 individuals. The estimates for ancestral population size show that *P. t. ellioti* north of the Sanaga River were approximately 6,000 individuals, and that the ancestral populations of chimpanzees likely ranged between approximately 3,000 and 7,000 individuals. The number of migrants per generation was also estimated for all three pairs of populations. Between 0.79 and 1.58 migrants per generation have been exchanged between populations across the Sanaga River, since the initial separation of *P. t. troglodytes* and *P. t. ellioti*. Higher levels of migration were found between the *P. t. ellioti* populations (Table 3 and Additional file 7).

**Table 3:** **Summary of demographic parameters for population pairs**

**Comparison**				Migrants per generation*		Population Size*			Population Divergence*
**Population 1**	**x**	**Population 2**		**Into Pop. 1**	**Into Pop. 2**	**Ne Pop. 1**	**N** ***e*** **Pop. 2**	**N** ***a***	**T**_**MRCA**_ **(kya)**
***P. t. ellioti***	**x**	***P. t.***	***MLE***	*1.10*	*1.15*	*2*,*140*	*3*,*365*	*3*,*002*	*200*,*979*
**(Rainforest)**		***troglodytes***	*95*% *CI*	*0.52* - *2.37*	*0.32* - *2.53*	*1*,*748* - *2*,*696*	*2*,*720* - *4*,*091*	*222* - *15*,*294*	*180*,*157* - *334*,*096*
***P. t. ellioti***	**x**	***P. t.***	***MLE***	*1.58*	*0.79*	*2*,*633*	*9*,*084*	*7*,*208*	*254*,*553*
**(Ecotone)**		***troglodytes***	*95*% *CI*	*1.03* - *2.34*	*0.20* - *1.71*	*1*,*984* - *3*,*282*	*7*,*549* - *10*,*961*	*1*,*493* - *45*,*421*	*202*,*149* - *328*,*715*
***P. t. ellioti***	**x**	***P. t. ellioti***	***MLE***	*2.49*	*4.39*	*2*,*368*	*860*	*5*,*920*	*4*,*219*
**(Rainforest)**		**(Ecotone)**	*95*% *CI*	*0.62* - *13.73*	*1.84* - *8.84*	*1*,*466* - *3*,*077*	*658* - *1*,*231*	*4*,*200* - *12*,*465*	*2*,*298* - *67*,*636*

*Demographic parameters were inferred in IMa [38]. These parameters were scaled assuming an mtDNA mutation rate of 1.64 x 10 ^– 7^ [89], an intermediate microsatellite mutation rate of 7.75 x 10 ^- 5^ [87], and 20-year generation time [90]. Demographic estimates assuming different microsatellite mutation rates are shown in Additional file 11.

Overall the results of the IMa suggest very clearly that the gene pools of *P. t. ellioti* and *P. t. troglodytes* are mostly, but not completely isolated from one another (Figures 3, 5 and 6). We conclude that these two subspecies are characterized by an isolation-with-migration population history model [40] for several reasons. The results of the IMa analysis shows that the *P. t. ellioti* populations last shared a common ancestor with *P. t. troglodytes* approximately 200–250 kya, and since this separation they have exchanged approximately one migrant per generation. Moderate levels of gene flow (≥1 migrant/generation) between populations should prevent divergence, under a model that assumes that drift occupies the dominant role in population differentiation [40]. Population divergence in the presence of gene flow is often cited as evidence that local adapatation is driving the separation of two or more populations [41-43]. However, the observed value of migration between *P. t. elioti* and *P. t. troglodytes* (2 Nm ~ 1) is a minimal threshold [40], making it difficult to fully distinguish whether allopatric speciation or local adaptation has occupied the dominant role in driving this separation.

Demographic histories can be influenced by climatic histories. The climate of Africa has been affected by oscillations in global temperature and species assemblages in tropical Africa are often influenced by these changes, particularly the Last Glacial Maximum (LGM) [25]. It is also important to understand demographic history in the context of climatic histories. Interestingly, the dates of divergence between *P. t. ellioti* and *P. t. troglodytes* predate the LGM, but do coincide with a pronounced glacial episode approximately 250kya [26], with evidence that sand dunes extended all the way to the Niger Delta [25].

It may be possible that these fluctuations in local climate may have influenced the Sanaga River, but little is known regarding its historical course and size. Rivers are known to dramatically change in course and size over time, thus affecting their ability to act as dispersal barriers [44]. Thus, it is possible that chimpanzee migration across the Sanaga could have occurred in bursts when river size was minimal, but we are unable to directly test this hypothesis, given the nature of the genetic data used in this study.

## Conclusions

This study represents the most comprehensive genetic dataset available for wild chimpanzees from Cameroon, which is important because it is the only known area where the distributions of two chimpanzee subspecies overlap. Microsatellite genotype profiles from 187 unrelated individuals and mtDNA haplotypes of 607 individuals were used to test between a variety of hypotheses for the presence and type of population structure of chimpanzees across the study area, including (*i*) panmixia, (*ii*) isolation-by-distance, (*iii*) population structure with complete isolation, and (*iv*) population structure with ongoing migration. Overall, the results suggest that *P. t. ellioti* and *P. t. troglodytes* represent genetically distinct populations in Cameroon, confirming results from previous studies of captive chimpanzee with inferred origins [6-8,39] and previous analyses of mtDNA haplotypes of wild individuals [9,10]. Surprisingly, we found additional evidence that *P. t. ellioti* consists of two genetically- and geographically-distinct populations. One population is located in forested regions of eastern Nigeria and western Cameroon and the second is located in central Cameroon, in a savanna-woodland mosaic that occurs between the Mbam and Sanaga Rivers. The results of the IMa analysis show that the *P. t. ellioti* populations and *P. t. troglodytes* diverged from one another 200–250 kya, and have experience moderately high (2Nm ~ 1) gene flow since (Table 3). This, coupled with the overlap of these populations in the mtDNA haplotype network analysis (Figure 3) and microsatellite cluster analyses (Figures 5 and 6) call in question the role of the Sanaga River in acting as a biogeographic barrier that has separated these chimpanzee subspecies.

Several riverine barriers have been proposed as biogeographic boundaries for primates in the region [4,9,12,14-18,45], but the role of habitat variation in driving primate speciation remains unknown. For instance, in addition to being important for chimpanzees, the Sanaga River has been proposed to influence the distribution of several pairs of primates, including *Mandrillus leucophaeus*/*M. sphinx*, *Cercopithecus erythrotis*/*C. cephus*, *C. nictitans martini*/*C. n. nictitans*, and *C. pogonias pogonias*/*C. p. grayi* [4,14-16,18]. These pairs of primates all occupy vastly different habitats and niches [14,15]. This observation suggests that other factors along with, or instead of, the Sanaga River may be important in separating the distribution of these species, subspecies and populations across the region. It is well documented that non-riverine barriers, such as the Dahomey Gap, also separate taxa, and likely separates *P. t. verus* from *P. t. ellioti* (although the role of the Niger River is unclear) [10,44]. Other factors have also been proposed to shape African tropical biodiversity in the region [12,46-49]. Thus, it is important that we reevaluate the role that the allopatric speciation, as driven by the Sanaga River, may have had in governing distributions of rainforest taxa, particularly primates.

Cameroon lies at the intersection of two major rainforest biomes. The Congolian Rainforest extends northward into southern Cameroon from central Africa, and the Guinean Rainforest extends southward into eastern Nigeria and western Cameroon from western Africa (Figure 1a). These two biomes converge in central Cameroon at the location of a pronounced ecotone [20], which is composed of open woodland, savannah and riparian forest [20,21]. Recent studies in this area that have combined genetic, morphological and environmental data have found that this ecotone appears to drive evolutionary diversification in insects [50], reptiles [51] and birds [22,52]. This is especially interesting, given that neutral processes are unlikely to explain the population history of chimpanzees in the region, as they seem to follow an isolation-with-migration model. The results of the mtDNA haplotype analysis shows an overlap of the two major haplogroups (Figure 3), and the results of the cluster analysis show a distinct deme of chimpanzee in this ecotone (Figure 6d). However, the loci included in this study meet expectations of neutral evolution, and thus, do not allow for us to draw additional conclusions regarding how ecological variation may drive diversification in chimpanzees. These observations underscore the importance of testing alternative hypotheses with loci that that are neutral *and* under selection.

Even though the migration rate between *P. t. ellioti* and *P. t. troglodytes* (2Nm ~ 1) is a minimal threshold to propose that selection has driven the separation of these populations, there is mounting evidence that allopatric speciation alone cannot account for the divergence of these populations. A complimentary study of the ecological niche differentiation in these populations shows that the demes of chimpanzees found in this study also occupy significantly different niches [53], suggesting that environmental variation contributes to driving the differentiation of these populations. The alternative hypothesis, that speciation in chimpanzees is driven by allopatric speciation, would be explained by an absence of niche divergence among these three demes [54,55]. Furthermore, in another complimentary study we found significant associations between genetic differentiation in these three demes and environmental variation across the study area [56], which led us to conclude that these populations likely follow a pattern of isolation-by-environment [57], a relationship between populations that arises as a result of local adaptation to different environments. Taken together, these findings are consistent with the observation that chimpanzees in Cameroon and Nigeria may be adapted to their local evironments, and that this variation has contributed to the genetic differentiation of chimpanzee subspecies.

A broader understanding about the role of local adaptation in chimpanzees and other taxa may provide important clues regarding why this region of Africa contains such a high proportion of the Earth’s biodiversity. Future studies that more closely examine the history of the Sanaga River and the role of historic climatic variation in shaping chimpanzee genetic diversity are crucial for building this understanding. Additionally, a better understanding of how chimpanzee social stuctures and dispersal patterns are shaped by habitat variation, and how this may contribute to regional genetic diversity, is of vital importance to unravelling the forces that have helped make this region such an incredibly diverse place on Earth.

## Methods

### Dataset preparation

We gathered fecal (n = 247) and hair samples (n = 223) from wild-living, non-habituated chimpanzee populations from 36 remote regions spanning eastern Nigeria through southern Cameroon (Figure 2 and Additional file 1). Most samples originating from the regions of Belgique (BQ), Boumba Bek (BB), Diang (DG), Dja Biosphere (DB), Duomo Pierre (DP), Ekom (EK), Lobeke (LB), Mambele (MB) and Minta (MT) were collected between 2004 and 2006 by the team of BHH and MP. Hair and tissue samples from Nigeria and Cameroon were collected during a series of studies from 1994 – 2010 by the team of MKG. We recruited a team of ~9 field assistants for each mission and base camps were established in the vicinity of forests were chimpanzee presence was reported by local hunters and villagers. We initially walked transects, hunters’ paths or elephants paths in search of chimpanzees presence (e.g. night nests, foot prints and vocalizations). The Campo Ma’an (CP), Cross River (CR), Deng Deng (DD), Diang (DG), Dja Biosphere (DB), Douala-Edea (DE), Gashaka Gumti (GG), Mbam et Djerem (MD) and Mount Cameroon (MC) sites are located in National Parks or forests reserves, whereas the remaining field sites are located in unprotected forests. Fecal samples were identified to be of likely chimpanzee origin by experienced trackers and/or by the researchers. Fecal samples (15–20 g) were placed into 30 or 50 mL tubes and mixed with equal amounts of RNA*later*® (Ambion, Austin, TX). Hair samples were collected directly from abandoned sleeping nests and a minimum of three hairs per nest were stored into glassine envelopes, and kept dry in silica gel. Fecal samples were inspected to estimate their likely time of deposition, whereas night nests age was estimated according to a leaf decay index [58]. Time, date, location, longitude, latitude and name of collector were also recorded. Fecal and hair samples were generally kept at ambient temperature for no longer than 2 weeks and subsequently stored at −20°C once back in Yaoundé, Cameroon. Samples were shipped to the United States at ambient temperature, then stored at −20°C upon receipt. All samples were transported from Cameroon to the United States in full compliance with Convention of International Trade in Endangered Species of Wild Fauna and Flora (CITES) and Center for Disease Control (CDC) export and import regulations. This research was carried out with IACUC approval from the University at Albany – State University of New York.

We extracted fecal DNA using the QIAamp Stool DNA Mini kits (Qiagen, Valencia, CA) following well-established protocols [59]. We briefly resuspended 1.5 mL of fecal RNA*later*® mixture in stool lysis buffer and clarified them by centrifugation. We treated the supernatants with an InhibitEx tablet, subjected them to proteinase K digestion, and passed them through a DNA binding column. Bound DNA was eluted in 100 μL elution buffer. We extracted DNA from hair samples using a chelating resin protocol [60] followed by filtration using Microcon 100 columns (Millipore, Billerica, MA) to concentrate DNA extracts. We conducted the preparation of the DNA quantification standards, reagents and reactions according to the Quantifiler® DNA Manufacturer’s protocol [61]. We analyzed the data using a 7500 System v1.2.3 software as an ‘Absolute Quantification (standard curve)’ assay with the settings recommended in the Quantifiler® Kit User’s Manual [61].

We used primers L15997 (5′-CACCATTAGCACCCAAAGCT-3') and H16498 (5′-CCTGAAGTAGGAACCAGATG-3′) to amplify a 460 – 500-bp mtDNA fragment spanning the hypervariable D-loop region (HVRI) in all samples that were newly collected for this study, using methods described in previous studies [10,13,59]. We assembled and aligned the resulting sequences with SEQMAN DNASTAR software (Lasergene, DNASTAR, Inc., Madison, WI), along with georeferenced sequences from previous studies [9,10,59,62]. We deposited all newly generated sequences from this study in GenBank under accession numbers KM401682-KM401815.

We used twenty-one autosomal microsatellite loci to produce genotype profiles from both hair and fecal samples. Additional file 14 lists information about the markers including the primers flanking the selected regions and the fluorescent dye set (Applied Biosystems, Foster City, CA) chosen. These markers were originally developed for use in humans [63], but have been shown to be highly informative for use in chimpanzee population genetics studies [39,64]. We also included the Amelogenin locus to determine the sex of the individual sampled. We divided these 22 loci into 4 multiplex PCR reactions that were performed using the Quiagen Multiplex PCR Kit (Qiagen, Valencia, CA) in Eppendorf Mastercyclers (Eppendorf, Westbury, NY). We used 0.5-1 ng DNA, along with Q-Solution (included in the kit). We adopted the following PCR conditions: an initialization at 95°C for 15 min, followed by 40 cycles including a denaturation step at 95°C for 30 sec, an annealing temperature of 60°C for 1 min, an elongation at 72°C for 1 min and 30 sec, and a final extension at 72°C for 10 min. For samples that failed to produce reliable genotype profiles, we increased the amount of DNA template to up to 8 μL per reaction, regardless of the DNA quantitation results. Many of the hair samples from the Nigerian locations had been typed previously for some of the loci selected, and were adjusted to the differences in base pair sizes due to apparatus and protocol discrepancies [13,65]. All PCR reactions included negative control samples for quality assurance. We analyzed each multiplex PCR product on an ABI 3130 capillary array genetic analyzer (Applied Biosystems, Foster City, CA). We determined fragment sizes against a Genescan 600 Liz size standard (Applied Biosystems, Foster City, CA), and allele sizes using the Genemapper ID version 2.7 software (Applied Biosystems, Foster City, CA). We scored alleles between three and 6 times to avoid problems associated with allelic dropout, which frequently occurs when genotyping low-yield DNA samples [66]. Samples that did not include at least 15 (70%) or more loci after multiple attempts at PCR fragment amplification were excluded from this study.

We subjected all 21 autosomal microsatellite loci to outlier tests using LOSITAN [67]. LOSITAN is a Java based selection detection platform, based on the fdist *F*_*ST*_ outlier methods [68]. We ran 1,000,000 simulations of the data while (*i*) assuming a stepwise mutation model, (*ii*) assuming three populations, (*iii*) forcing a correct mean *F*_*ST*_, and (*iv*) calculating a “neutral” mean *F*_*ST*_. We also subjected the 21 autosomal microsatellites, in addition to the mtDNA HVRI sequences, to an exact test of Hardy-Weinberg equilibrium [69] using the Arlequin version 3.5 software package [37]. We tested deviations from Hardy-Weinberg equilibrium for each locus against 1,000,000 random permutations of the data.

We calculated pairwise estimates of relatedness for all samples that produced reliable microsatellite genotypes using the software program COANCESTRY [23]. COANCESTRY implements three inbreeding estimators and seven relatedness estimators [24,70-76] to estimate relatedness between individuals and inbreeding coefficients from multi-locus genotype data. We tested 95% confidence intervals for relatedness and inbreeding estimates for all genotyped individuals against 1000 bootstrap permutations of the data. Samples estimated to come from the same individual or close relatives, with a relatedness index [24] above 0.75, were excluded in order to remove all pairs of individuals down to first cousins from all further data analyses.

### mtDNA diversity analysis

We generated haplotype networks for HVRI mtDNA sequences via the median-joining algorithm of Network 4.5 (http://www.fluxus-engineering.com). Because it allows for reticulation, the median-joining approach for inferring haplotype relationships is appropriate for the analyses of mtDNA control region sequences, which exhibits high levels of homoplasy in humans [77,78]. We identified hypermutable sites by post-processing using the Steiner maximum parsimony algorithm within Network 4.5 and were excluded from the network analyses.

We performed an AMOVA using Arlequin version 3.5 software package [37]. We tested population differentiation values against 10,000 random permutations of the data. For the AMOVA, we grouped individuals according to their origin either north or south of the Sanaga River. In addition to the AMOVA, we ran a spatial AMOVA (SAMOVA) at *k* = 2, to confirm the groupings used in the AMOVA, as well as to test how geospatial partitioning of the sampled populations affected population differentiation [28].

We generated mismatch distributions by computing distribution of the number of pairwise differences between mtDNA haplotypes using Arlequin version 3.5 software package [37]. We generated distributions by grouping individuals into either two or three separate groups (as identified by microsatellite cluster analysis), using 100 bootstrap replicates.

### Microsatellite genotype analysis

We examined the relationship between genetic differentiation and geographic distance by carrying out partial Mantel tests on the microsatellite data using the Arlequin version 3.5 software package [37]. Pairwise *F*_*ST*_ values were generated between all sampling locations and genetic differentiation was plotted against straight-line, geographic distance. We fit the data with a linear regression, calculated a correlation coefficient and tested for statistical significance.

We used the EIGENSOFT software package [79] to perform PCA on individual genotypes to identify individuals that could be grouped into significantly different populations. We converted microsatellite data into a false SNP format by scoring the presence or absence of each of n-1 alleles (where n is the number of alleles in the sample) using a script in MATLAB (The MathWorks, Natick, MA) described previously [39]. We processed this file in SmartPCA, which produced eigenvectors and eigenvalues. We conducted this analysis blindly to *a priori* population labels for individuals in the dataset. We tested the statistical significance of each eigenvector using Tracy-Widom statistics. Each significant eigenvector recovered by this PCA approach separates the samples in such a way that the first and subsequent eigenvectors distinguish, in order, the most to least differentiated populations in the sample [79]. All analyses using EIGENSOFT were performed blinded to a priori population labels.

We examined population structure and individual ancestry using a Bayesian clustering approach implemented in the TESS (version 2.3) software package [30]. This program estimates the shared population history of individuals based on their genotypes and geographic origin under a model that assumes Hardy–Weinberg equilibrium and linkage equilibrium, thereby making no *a priori* assumptions regarding population classifications. TESS estimates individual proportions of ancestry into K clusters, where K is specified for the program in advance across independent runs and corresponds to the number of putative ancestral populations. The program then assigns admixture estimates for each individual (*Q*) from each inferred ancestral population cluster. It also produces posterior predictive values of *Q* for geographic areas located in between and around the individual data points.

TESS runs were performed: (*i*) with a model that allows individuals to have ancestry in multiple populations (CAR model [80]); (*ii*) with correlated allele frequencies; and (*iii*) blinded to *a priori* population labels. Runs were performed with a burn-in step of 500,000 Markov Chain Monte Carlo (MCMC) iterations and 1,000,000 MCMC iterations. Fifty runs each for K = 2 through K = 8 were performed for all datasets. We processed TESS outputs with CLUMPP [81] and a G-statistic >99% was used to assign groups of runs to a common clustering pattern. CLUMPP outputs for each K value were plotted with DISTRUCT [82]. We used a combination of methods to infer a maximum number of chimpanzee populations (*K*_*MAX*_) including, (*i*) the K value that had the highest average DIC [31], (*ii*) high stability of clustering patterns between runs, (*iii*) the *K*_*MAX*_ value at which *K*_*MAX*_ + 1 no longer split the cluster distinguished by *K*_*MAX*_ [83], (*iv*) correspondence between maximum PPD values from TESS runs and significant eigenvectors recovered by PCA, and (*v*) calculating an *ad hoc* statistic, ΔK [32].

We created spatial interpolations of the *Q* matrix using the option in TESS to generate posterior predictive maps of admixture proportions. We created a template map of the extent of probable chimpanzee ranges in Nigeria and Cameroon in ArcMap Version 10 (ESRI Corp., Redlands, CA) in the ASCII-raster format and input into TESS in order to generate posterior predictive maps of admixture proportions. Using these predictive maps, we generated spatial interpolations of the *Q* matrices by implementing a matrix based vector algorithm in the program TESS Ad-Mixer [33]. These spatial interpolations use color mixing to predict admixture proportions across space in order to better visualize the spatial partitioning of genetic differentiation.

We calculated allele size range, observed and expected heterozygosity, and an M-Ratio [84] for each locus for three populations as identified by cluster analysis. We tested these values against 10,000 random permutations of the data using the Arlequin version 3.5 software package [37].

We calculated three measures of population genetic differentiation using the Arlequin version 3.5 software package [37]: *D*^*2*^, *R*_*ST*_ and *δμ*^*2*^. The *D*^*2*^ [34] genetic distance is based on a model in which genetic drift is the only force influencing allele frequency differences across populations and is sensitive to recent differentiation events. *R*_*ST*_ [35] and *δμ*^*2*^ [36] are similar to *D*^*2*^, but both assume a stepwise mutation model (SMM). Consequently, *R*_*ST*_ and *δμ*^*2*^ are more likely to capture whether differences in the mutation processes are important in driving population differentiation and enable assessment of sensitivity to mutation model assumptions [83]. These latter models differ in that *R*_*ST*_ is based on the fraction of the total variance in allele size between populations and is analogous to *F*_*ST*_ [35], whereas *δμ*^*2*^ is based on differences in the means of microsatellite allele sizes [36]. Recent work has shown convincing evidence that the loci typed for this study appear to follow the SMM in both chimpanzees and bonobos [64,85]. *D*^*2*^ calculations were completed on untransformed allele size calls. Since *R*_*ST*_ and *δμ*^*2*^ assume the SMM, we transformed allele sizes to repeat size units prior to analysis in Arlequin version 3.5 [37]. We transformed allele sizes such that the smallest allele for each locus was scored as n and each subsequent allele was scored as n + 1. In infrequent cases where repeat unit sizes did not follow the n + 1 model, and instead repeat units skipped x repeat(s), we scored the next allele in the data as (n + × +1), as described in a previous study [39]. We determined each pairwise genetic distance calculation by 100,000 replications in Arlequin, and the significance of these pairwise population genetic distances were evaluated by a significance test at p < 0.05.

We calculated distributions of alleles within and between populations using ADZE [86]. ADZE is a generalized rarefaction approach that counts alleles private to populations as well as combinations of populations. We used this program to infer (*i*) the mean number of distinct alleles per locus, per population; (*ii*) the mean number of private alleles per locus, per population; and (*iii*) the mean number of uniquely shared private alleles per locus, between populations.

### Population history

We used the program IMa [38] to estimate: (*i*) the population mutation parameter, θ, for both descendant populations (θ_1_ and θ_2_) and an ancestral population (θ_A_); (*ii*) rates of migration from population 1 to population 2(*m*_1_) and from population 2 to population 1 (*m*_2_); (*iii*) and time of population divergence (*t*) for all possible pairs of the three assumed populations: *P. t. ellioti* in western Cameroon & Nigeria, *P. t. ellioti* in central Cameroon, and *P. t. troglodytes* in southern Cameroon. We used IMa instead of the more recently released IMa2 [40] for several reasons. The dataset is composed mostly of microsatellites with poorly characterized mutation rates [39,64,87]. Also, due to the complexity of the IMa2 model, the high number of microsatellite alleles, and few number of microsatellite loci, we used the version of the IM model with the fewest assumptions. Additionaly, IMa2 requires the user to input a well resolved, rooted phylogeny of the populations to be tested, and based on the results of the cluster analysis, it was counterproductive to swap through an already well resolved, and simple phylogeny.

We ran multiple iterations of IMa in parallel, for each paired population, using a multi-processor computer cluster located at the SUNY College of Nanoscale Science and Engineering. First, we ran the IMa analysis using “M-Mode” (MCMC Mode) with a full complement of model parameters, and a broad range of priors for all parameters (θ_1_, θ_2_, θ_A_, *m*_1_*m*_2_, and *t*). Each run was performed with heated chains using the two-step scheme [88]. For each run, we ran burn-in replicates for a total of five days. We then reduced the ranges of the model parameters over repeated runs in order to sample more densely their respective posterior distributions for each of the three population comparisons. This resulted in different lengths of analysis for each comparison: the western Cameroon/central Cameroon comparison generated 1,004,512 genealogies using 58 chains; the western Cameroon/southern Cameroon comparison generated 684,192 genealogies using 40 chains; and the central Cameroon/southern Cameroon comparison generated 947,723 genealogies using 38 chains. After the runs converged toward the same values, we loaded the saved genealogies from multiple runs into “L-Mode” (Load Trees Mode) in order to calculate values for model parameters for each pair of populations pooled across these multiple independent runs.

We converted the migration parameters, *m*_1_ and *m*_2_, into the number of migrants exchanged per generation (*Nm*) using the equation *Nm* = (θ*m)/2. We converted the demographic parameter (*t*) into years by scaling by mutation rate (*t*/μ). We scaled the *t* parameter for mtDNA assuming a mutation rate (μ) of 1.64 × 10^−7^ [89]. We scaled the *t* parameter for microsatellite loci using several microsatellite mutation rates, given the uncertainty in microsatellite mutation rates in the literature [36,39,64,87]. We used a slow mutation rate of 3.53 × 10^−5^ [64], an intermediate mutation rate of 7.75 × 10^−5^ (calculated from the geometric mean of rates from Wegmann and Excoffier [87]), and a fast mutation rate of 1.6 × 10^−4^ [36,39]. We scaled final values for demographic parameters assuming a 20-year generation time for chimpanzees [90]. We scaled effective population sizes (*N*_*E*_) using θ and a per generation μ using the equation *N*_*E*_ = θ/(4*μ*20).

## Additional files

Additional file 1:
**Sample locations and number of individuals included in study.** ^a^Individuals determined by relatedness indices calculated using COANCESTRY [23]. ^b^Samples from these locations were not genotyped for microsatellite loci. Additional file 2:
**Summary Statistics for mtDNA HVRI.** ^a^Significant values of Fu’s FS shown as bold. ^b^p = 0.04. ^c^p < 0.001. Additional file 3:
**Summary statistics for microsatellite loci.** *Observed heterozygosity calculated in Arlequin [37]. †Expected heterozygosity calculated in Arlequin [37]. ‡M-Ratio from Garza & Williamson [84]. Additional file 4:
**Identification of neutral versus outlier loci.** Heterozygosity was plotted against *F*_*ST*_ for all 21 microsatellite loci using LOSITAN [67]. Ranges of values for balancing selection (yellow), positive selection (red) and neutrality (gray) were identified. All 21 microsatellite loci fell into the acceptable range of neutrality. Additional file 5:
**Spatial Analysis of Molecular Variance (SAMOVA) for mtDNA HVRI.**
Additional file 6:
**Mismatch distribution for two populations.** Mismatch distribution using the mtDNA HVRI locus for *P. t. ellioti* (A) and *P. t. troglodytes* (B). Harpending’s Raggedness Index for *P. t. ellioti* (A) was 0.003 (p = 0.977), and for *P. t. troglodytes* (B) it was 0.007 (p = 0.168). Additional file 7:
**Mismatch distribution for three populations.** Mismatch distribution using the mtDNA HVRI locus for *P. t. ellioti* (Rainforest, A), *P. t. ellioti* (Ecotone, B) and *P. t. troglodytes* (C). Harpending’s Raggedness Index for *P. t. ellioti* (Rainforest, A) was 0.002 (p = 0.989), for *P. t. ellioti* (Ecotone, B) it was 0.008 (p = 1) and for *P. t. troglodytes* (C) it was 0.007 (p = 0.168). Model frequency for *P. t. ellioti* (Ecotone, B) was not plotted, because the analysis only generated a value for one sample point. Additional file 8:
**Estimating** ***K***_***MAX.***_ *K*_*MAX*_ was inferred from 50 independent TESS [30] runs for each value of *K* from 1 to 8. (A) Estimated values for the Deviance Information Criteria (DIC) [31]. (B) Estimated Δ*K* [32] values. Additional file 9:
**Genetic differentiation (** ***D*** ^***2***^ ***,R***_***ST***_ **and** ***δμ*** ^***2***^ **) and migration (2N** ***m*** **) between populations from across the study area from 21 autosomal microsatellite loci.** ^a^Measures of differentiation were computed between populations as inferred by the cluster analyses. ^b^Values above the diagonal are based on Reynold’s Coancestry Coefficient, *D* ^*2*^, which does not assume that mutations follow the SMM [34]. ^c^Values in underlined italics are migrants exchanged between populations each generation (2N*m*). ^d^Values below the diagonal are based on an *R*_*ST*_ [35] model of evolution that assumes that mutations follow the SMM. ^e^Values in parentheses are based on *δμ* ^*2*^ model of evolution that also assumes that mutations follow the SMM [36]. ^f^All population pairwise values were significantly different from null expectations from 10,000 permutations of the data in Alrequin [37]. Additional file 10:
**Inference of microsatellite allelic diversity.** (A) Mean number of distinct alleles found in sampled populations. (B) Mean number of private alleles found in sampled populations. (C) Mean number of uniquely shared alleles between sampled populations. Additional file 11:
**Summary of results from IMa analysis assuming slow, medium, and fast mutations rates for microsatellite loci.** *Demographic parameters were scaled assuming a mtDNA mutation rate of 1.64 x 10^−7^ [89]. There is a great deal of uncertainty in microsatellite mutation rates. Thus, we scaled demographic parameter estimates using slow, intermediate and fast microsatellite mutation rates. Green cell show a demographic estimates calculated using a slow rate of 3.53 x 10^−5^ from Becquet *et al*. [64]. Blue cells show demographic estimates using an intermediate rate of 7.75 x 10^−5^ calculated from the geometric mean of rates from Wegmann and Excoffier [87]. Red cells show demographic estimates scaled with the fastest mutation rate of 1.6 x 10^−4^ [36,39]. Demographic parameters were scaled assuming a generation time of 20 years for chimpanzees [90]. Additional file 12:
**Tests of nested models from IMa for three populations of chimpanzees.** ^a^Description of model tested. ^b^LLR test statistics are calculated as twice the difference in log-likelihood between the indicated model and the full model (Θ_1_ Θ_2_ Θ_A_ m_1_ m_2_), with degrees of freedom equal to the difference in the number of parameters free to be estimated from the data. ^c^ *P* values are obtained by comparison to the Chi-square distribution. ^d^Number of free parameters, or degrees of freedom for each model. ^e^When the null model is true and has a parameter fixed at the boundary of the parameter space, the expected distribution is a mixture [38]. ^f^Bold numbers indicate models that could not be rejected (*p* > 0.05) by the 2LLR log-likelihood ratio test. Additional file 13:
**IMa probability distributions.** Posterior probability distributions for three parameters: (*i*) migration (2N*m*); (*ii*) effective population size (4Ne*μ*); and time of population divergence (T_MRCA_). Parameters are not scaled according to a mutation rate or chimpanzee generation time. Parameters were tested for three population comparisons: (A) *P. t. ellioti* (Rainforest) – *P. t. troglodytes*, (B) *P. t. ellioti* (Ecotone) – *P. t. troglodytes*, and (C) *P. t. ellioti* (Rainforest) – *P. t. ellioti* (Ecotone). Additional file 14:
**Microsatellite loci information.** ^a^Polymorphic Information Content estimated in CERVUS version 3.0 [91].
