# Supplementary material for: The population genetics of wild chimpanzees in Cameroon and Nigeria suggests a positive role for selection in the evolution of chimpanzee subspecies
Source: BMC Evol Biol. 2015 Jan 21;15:3. doi: 10.1186/s12862-014-0276-y (PMC4314757; doi:10.1186/s12862-014-0276-y)
Supplement: Additional file 1: — Sample locations and number of individuals included in study. aIndividuals determined by relatedness indices calculated using COANCESTRY [23]. bSamples from these locations were not genotyped for microsatellite loci. [file 12862_2014_276_MOESM1_ESM.docx]

| **Sampling Location** | **Number of samples collected** | **mtDNA** | **Microsatellites** | | | |
| --- | --- | --- | --- | --- | --- | --- |
|  |  | **Number of sequences included** | **100% genotyping (21 loci)** | **100-70% genotyping** | **Total samples genotyped (>70%)** | **Number of indivi-duals included^a^** |
| Akoh-Zanto (NGAZ) | 11 | 8 | 0 | 4 | 4 | 4 |
| Cross River (NGCR) | 10 | 6 | 0 | 0 | 0 | 0 |
| Gashaka Gumti (NGGG) | 16 | 10 | 5 | 5 | 10 | 10 |
| Ngel Nyaki (NGNN) | 5 | 4 | 0 | 0 | 0 | 0 |
| Ebo Forest (CMEB) | 25 | 15 | 9 | 10 | 19 | 19 |
| Liabelem Highlands (CMED) | 6 | 5 | 3 | 0 | 3 | 3 |
| Banyang-Mbo (CMBM) | 5 | 4 | 0 | 0 | 0 | 0 |
| Mone (CMYW) | 4 | 2 | 1 | 3 | 4 | 4 |
| Mosse (CMMV) | 12 | 9 | 1 | 1 | 2 | 2 |
| Mount Cameroon (CMMC) | 11 | 9 | 4 | 5 | 9 | 9 |
| Bankim (CMBK) | 5 | 3 | 1 | 1 | 2 | 2 |
| Deuk (CMDK) | 7 | 4 | 0 | 3 | 3 | 3 |
| Kombe (CMKM) | 18 | 16 | 6 | 1 | 7 | 7 |
| Linte (CMLN) | 16 | 9 | 0 | 5 | 5 | 5 |
| Makombe (CMMK) | 11 | 3 | 2 | 1 | 3 | 3 |
| Manb'ra (CMMB) | 14 | 0 | 0 | 4 | 4 | 4 |
| Mbam et Djerem (CMMD) | 16 | 15 | 3 | 6 | 9 | 9 |
| Mount Golep (CMMG) | 23 | 23 | 11 | 6 | 17 | 15 |
| Ngambe-Tikar (CMNT) | 12 | 10 | 0 | 5 | 5 | 5 |
| Vome (CMVM) | 13 | 7 | 3 | 3 | 6 | 6 |
| Wassa Emtse (CMWE) | 25 | 25 | 0 | 2 | 2 | 2 |
| Wouchaba (CMWC) | 10 | 10 | 5 | 1 | 6 | 6 |
| Yagba (CMYB) | 17 | 13 | 10 | 4 | 14 | 14 |
| Belgique (CMBQ) | 82 | 82 | 3 | 6 | 9 | 9 |
| Biwali (CMBI) | 2 | 2 | 1 | 1 | 2 | 2 |
| Boumba Bek (CMBB) | 29 | 29 | 0 | 6 | 6 | 6 |
| Campo Ma'an (CMCP) | 28 | 28 | 0 | 3 | 3 | 3 |
| Deng Deng (CMDD) | 10 | 5 | 1 | 3 | 4 | 4 |
| Diang (CMDG) | 29 | 29 | 0 | 8 | 8 | 8 |
| Dja Biosphere (CMDB) | 13 | 10 | 1 | 2 | 3 | 3 |
| Douala-Edea (CMDE) | 13 | 7 | 1 | 3 | 4 | 4 |
| Duomo Pierre (CMDP)^b^ | 82 | 82 | - | - | - | - |
| Ekom (CMEK)^b^ | 17 | 17 | - | - | - | - |
| Lobeke (CMLB)^b^ | 11 | 11 | - | - | - | - |
| Mambele (CMMB)^b^ | 18 | 18 | - | - | - | - |
| Minta (CMMT) | 74 | 74 | 7 | 10 | 17 | 16 |
| **Total** | **700** | **604** | **76** | **112** | **190** | **187** |
