# Supplementary material for: The population genetics of wild chimpanzees in Cameroon and Nigeria suggests a positive role for selection in the evolution of chimpanzee subspecies
Source: BMC Evol Biol. 2015 Jan 21;15:3. doi: 10.1186/s12862-014-0276-y (PMC4314757; doi:10.1186/s12862-014-0276-y)
Supplement: Additional file 2: — Summary Statistics for mtDNA HVRI. aSignificant values of Fu’s FS shown as bold. bp = 0.04. cp < 0.001. [file 12862_2014_276_MOESM2_ESM.docx]

|  | ***θ(Hom)*** | ***θ(k)*** | ***θ(S)*** | ***θ(π)*** | ***Tajima's D*** | ***Fu's FS*^a^** |
| --- | --- | --- | --- | --- | --- | --- |
| **2-Population Model** | | | | | | |
| *P. t. ellioti* | 28.73294 | 66.28345 | 18.57499 | 63.07154 | 6.91284 | **-0.41307^b^** |
| *P. t. troglodytes* | 31.97737 | 45.1402 | 19.31347 | 76.53778 | 8.71967 | 11.7904 |
| **3-Population Model** | | | | | | |
| *P. t. ellioti* (Rainforest) | 156.7999 | 185.97161 | 21.86955 | 67.65884 | 6.1161 | -**7.07407^c^** |
| *P. t. ellioti* (Ecotone) | 12.76177 | 17.75134 | 11.81637 | 57.7257 | 11.90336 | 21.28628 |
| *P. t. troglodytes* | 31.97737 | 45.1402 | 19.31347 | 76.53778 | 8.71967 | 11.7904 |
