# Supplementary material for: The population genetics of wild chimpanzees in Cameroon and Nigeria suggests a positive role for selection in the evolution of chimpanzee subspecies
Source: BMC Evol Biol. 2015 Jan 21;15:3. doi: 10.1186/s12862-014-0276-y (PMC4314757; doi:10.1186/s12862-014-0276-y)
Supplement: Additional file 3: — Summary statistics for microsatellite loci. *Observed heterozygosity calculated in Arlequin [37]. †Expected heterozygosity calculated in Arlequin [37]. ‡M-Ratio from Garza & Williamson [84]. [file 12862_2014_276_MOESM3_ESM.docx]

| *P. t. ellioti* (Rainforest) | | | | | | *P. t. ellioti* (Ecotone) | | | | | *P. t. troglodytes* | | | | |
| --- | --- | --- | --- | --- | --- | --- | --- | --- | --- | --- | --- | --- | --- | --- | --- |
| Locus | N | H_O_* | H_E_† | Allelic  Range | M-Ratio‡ | N | H_O_ | H_E_ | Allelic  Range | M-Ratio | N | H_O_ | H_E_ | Allelic  Range | M-Ratio |
| 1 | 100 | 0.66 | 0.88 | 14 | 0.93 | 160 | 0.76 | 0.87 | 14 | 0.87 | 106 | 0.79 | 0.86 | 12 | 1.00 |
| 2 | 100 | 0.72 | 0.84 | 11 | 0.83 | 156 | 0.74 | 0.84 | 7 | 1.00 | 106 | 0.85 | 0.87 | 12 | 0.92 |
| 3 | 92 | 0.72 | 0.86 | 11 | 0.92 | 138 | 0.57 | 0.84 | 21 | 0.50 | 98 | 0.76 | 0.87 | 14 | 0.73 |
| 4 | 102 | 0.24 | 0.33 | 8 | 0.67 | 158 | 0.72 | 0.75 | 8 | 1.00 | 112 | 0.88 | 0.87 | 11 | 0.92 |
| 5 | 88 | 0.43 | 0.84 | 12 | 0.77 | 130 | 0.25 | 0.79 | 15 | 0.63 | 78 | 0.33 | 0.74 | 6 | 1.00 |
| 6 | 86 | 0.58 | 0.62 | 5 | 1.00 | 146 | 0.42 | 0.67 | 5 | 1.00 | 62 | 0.61 | 0.81 | 7 | 0.88 |
| 7 | 96 | 0.67 | 0.90 | 24 | 0.60 | 155 | 0.66 | 0.85 | 15 | 0.81 | 92 | 0.72 | 0.81 | 12 | 0.85 |
| 8 | 98 | 0.67 | 0.83 | 11 | 1.00 | 160 | 0.55 | 0.84 | 10 | 1.00 | 110 | 0.49 | 0.78 | 9 | 0.80 |
| 9 | 100 | 0.60 | 0.88 | 17 | 0.78 | 158 | 0.65 | 0.74 | 17 | 0.67 | 98 | 0.57 | 0.92 | 24 | 0.76 |
| 10 | 102 | 0.51 | 0.55 | 9 | 0.80 | 162 | 0.49 | 0.51 | 8 | 1.00 | 110 | 0.75 | 0.86 | 10 | 1.00 |
| 11 | 88 | 0.55 | 0.78 | 6 | 1.00 | 134 | 0.49 | 0.81 | 16 | 0.59 | 108 | 0.80 | 0.87 | 13 | 1.00 |
| 12 | 94 | 0.72 | 0.93 | 20 | 0.86 | 148 | 0.69 | 0.93 | 22 | 0.87 | 98 | 0.73 | 0.93 | 22 | 0.87 |
| 13 | 84 | 0.50 | 0.83 | 8 | 0.78 | 146 | 0.52 | 0.79 | 8 | 0.89 | 98 | 0.53 | 0.86 | 9 | 0.90 |
| 14 | 96 | 0.73 | 0.72 | 6 | 0.86 | 160 | 0.75 | 0.66 | 6 | 1.00 | 108 | 0.70 | 0.68 | 7 | 1.00 |
| 15 | 90 | 0.73 | 0.86 | 13 | 0.93 | 140 | 0.64 | 0.85 | 18 | 0.74 | 94 | 0.79 | 0.88 | 19 | 0.70 |
| 16 | 90 | 0.62 | 0.72 | 7 | 0.88 | 138 | 0.57 | 0.74 | 7 | 1.00 | 106 | 0.77 | 0.80 | 7 | 1.00 |
| 17 | 94 | 0.66 | 0.83 | 10 | 1.00 | 154 | 0.66 | 0.76 | 11 | 0.75 | 104 | 0.81 | 0.80 | 9 | 1.00 |
| 18 | 96 | 0.48 | 0.59 | 6 | 0.86 | 152 | 0.47 | 0.73 | 4 | 1.00 | 104 | 0.67 | 0.79 | 7 | 1.00 |
| 19 | 90 | 0.62 | 0.75 | 7 | 0.75 | 142 | 0.68 | 0.64 | 12 | 0.62 | 110 | 0.73 | 0.84 | 8 | 1.00 |
| 20 | 84 | 0.76 | 0.89 | 21 | 0.59 | 146 | 0.67 | 0.85 | 13 | 0.86 | 106 | 0.91 | 0.92 | 21 | 0.73 |
| 21 | 88 | 0.59 | 0.74 | 7 | 0.50 | 154 | 0.73 | 0.78 | 7 | 0.75 | 100 | 0.64 | 0.69 | 5 | 0.83 |
| μ | 93.24 | 0.61 | 0.77 | 11.10 | 0.82 | 149.38 | 0.60 | 0.77 | 11.62 | 0.83 | 100.38 | 0.71 | 0.83 | 11.62 | 0.90 |
| σ^2^ | 5.81 | 0.13 | 0.14 | 5.39 | 0.14 | 9.54 | 0.13 | 0.10 | 5.27 | 0.16 | 11.81 | 0.14 | 0.07 | 5.52 | 0.11 |
