# Supplementary figures and images for: The population genetics of wild chimpanzees in Cameroon and Nigeria suggests a positive role for selection in the evolution of chimpanzee subspecies
Source: BMC Evol Biol. 2015 Jan 21;15:3. doi: 10.1186/s12862-014-0276-y (PMC4314757; doi:10.1186/s12862-014-0276-y)

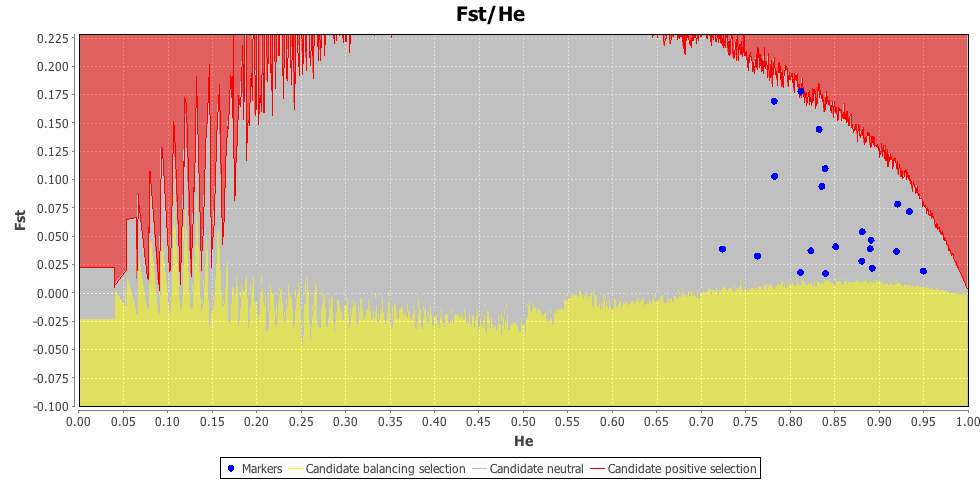

Supplement: Additional file 4: — Identification of neutral versus outlier loci. Heterozygosity was plotted against F ST for all 21 microsatellite loci using LOSITAN [67]. Ranges of values for balancing selection (yellow), positive selection (red) and neutrality (gray) were identified. All 21 microsatellite loci fell into the acceptable range of neutrality. [file 12862_2014_276_MOESM4_ESM.png]

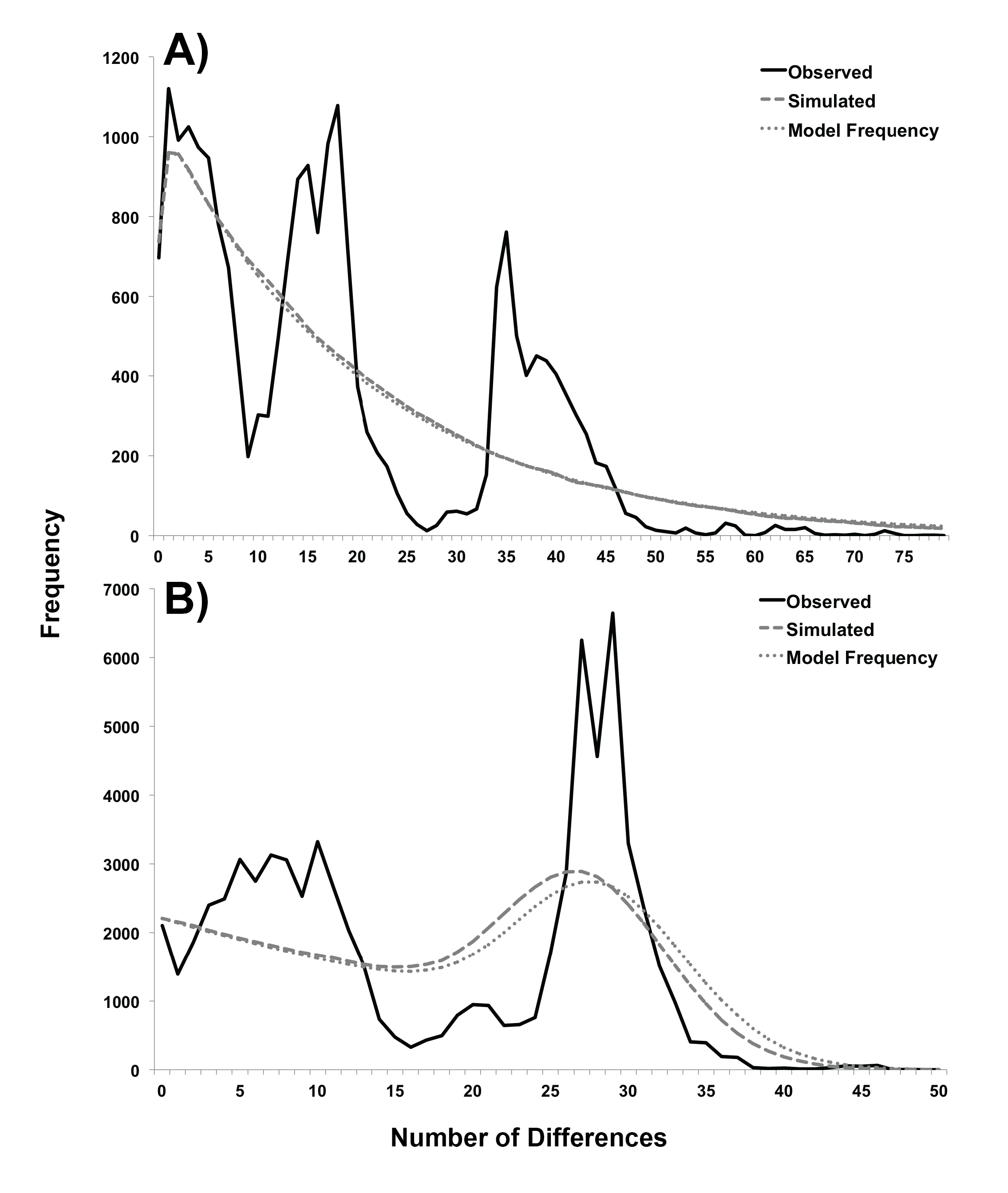

Supplement: Additional file 6: — Mismatch distribution for two populations. Mismatch distribution using the mtDNA HVRI locus for P. t. ellioti (A) and P. t. troglodytes (B). Harpending’s Raggedness Index for P. t. ellioti (A) was 0.003 (p = 0.977), and for P. t. troglodytes (B) it was 0.007 (p = 0.168). [file 12862_2014_276_MOESM6_ESM.png]

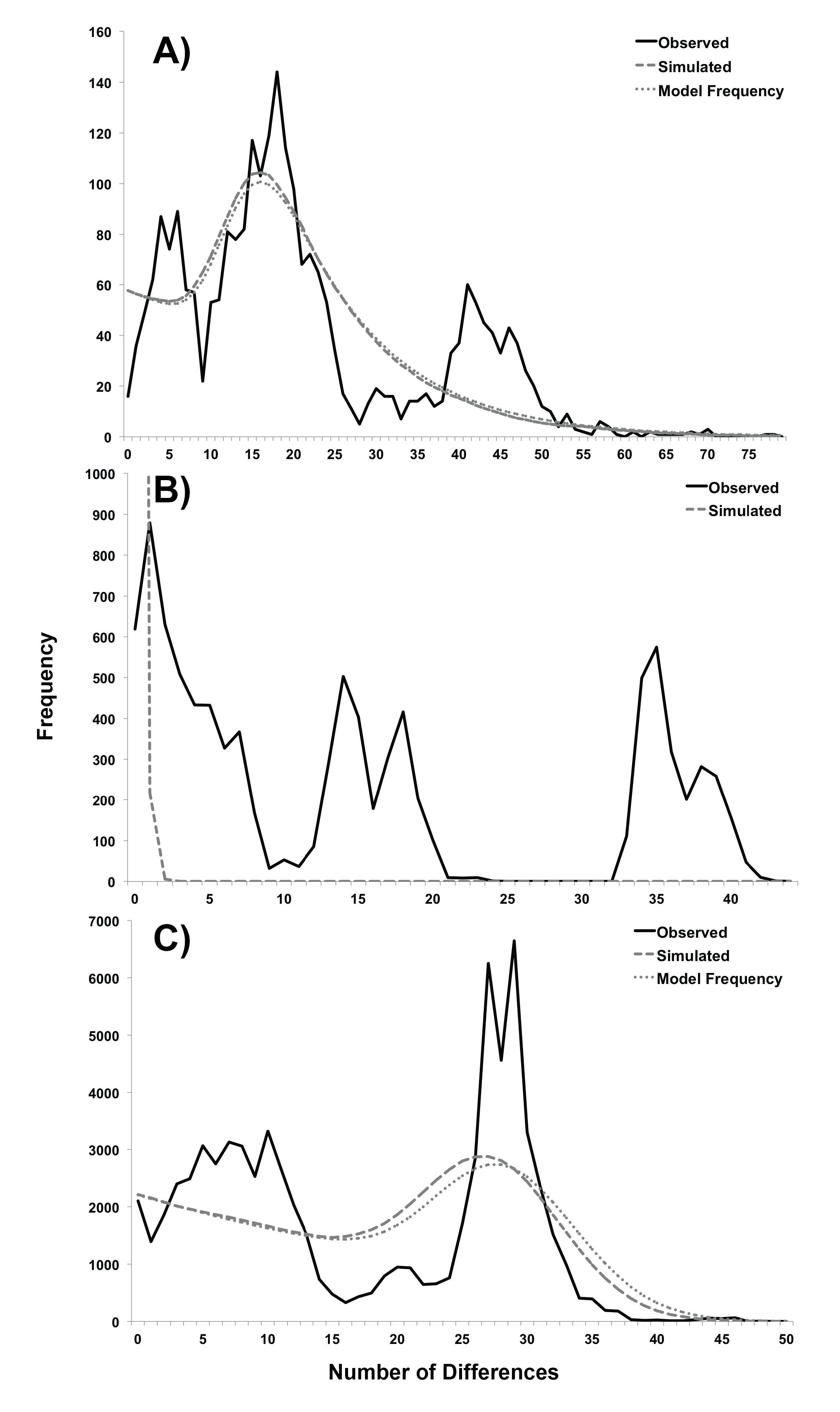

Supplement: Additional file 7: — Mismatch distribution for three populations. Mismatch distribution using the mtDNA HVRI locus for P. t. ellioti (Rainforest, A), P. t. ellioti (Ecotone, B) and P. t. troglodytes (C). Harpending’s Raggedness Index for P. t. ellioti (Rainforest, A) was 0.002 (p = 0.989), for P. t. ellioti (Ecotone, B) it was 0.008 (p = 1) and for P. t. troglodytes (C) it was 0.007 (p = 0.168). Model frequency for P. t. ellioti (Ecotone, B) was not plotted, because the analysis only generated a value for one sample point. [file 12862_2014_276_MOESM7_ESM.png]

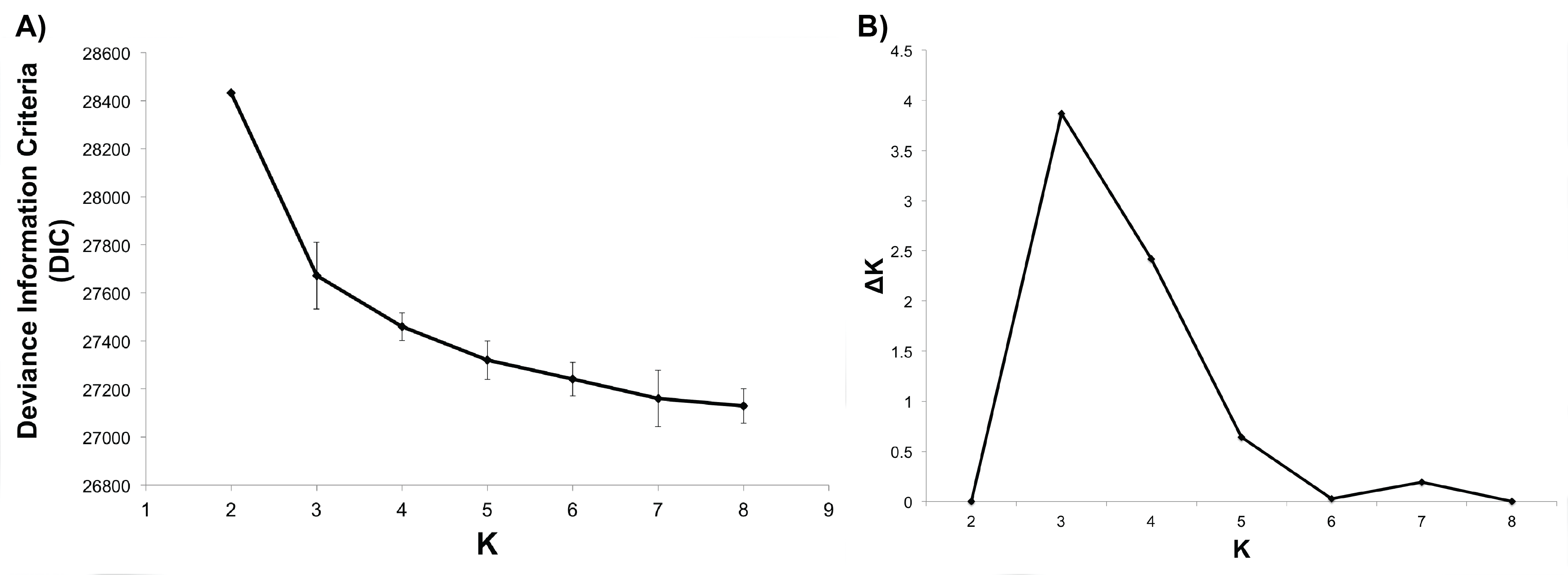

Supplement: Additional file 8: — Estimating K MAX. K MAX was inferred from 50 independent TESS [30] runs for each value of K from 1 to 8. (A) Estimated values for the Deviance Information Criteria (DIC) [31]. (B) Estimated ΔK [32] values. [file 12862_2014_276_MOESM8_ESM.png]

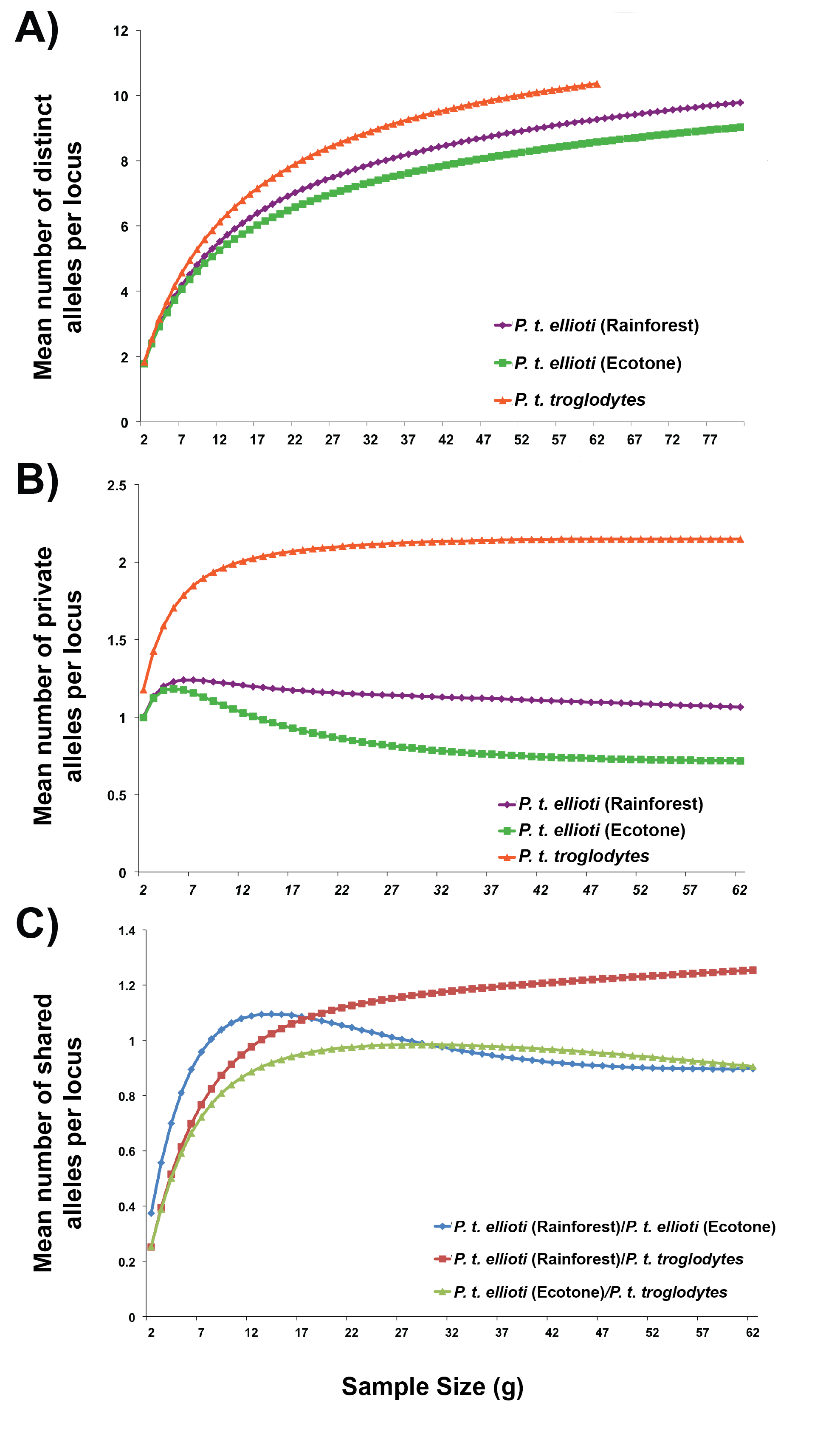

Supplement: Additional file 10: — Inference of microsatellite allelic diversity. (A) Mean number of distinct alleles found in sampled populations. (B) Mean number of private alleles found in sampled populations. (C) Mean number of uniquely shared alleles between sampled populations. [file 12862_2014_276_MOESM10_ESM.png]

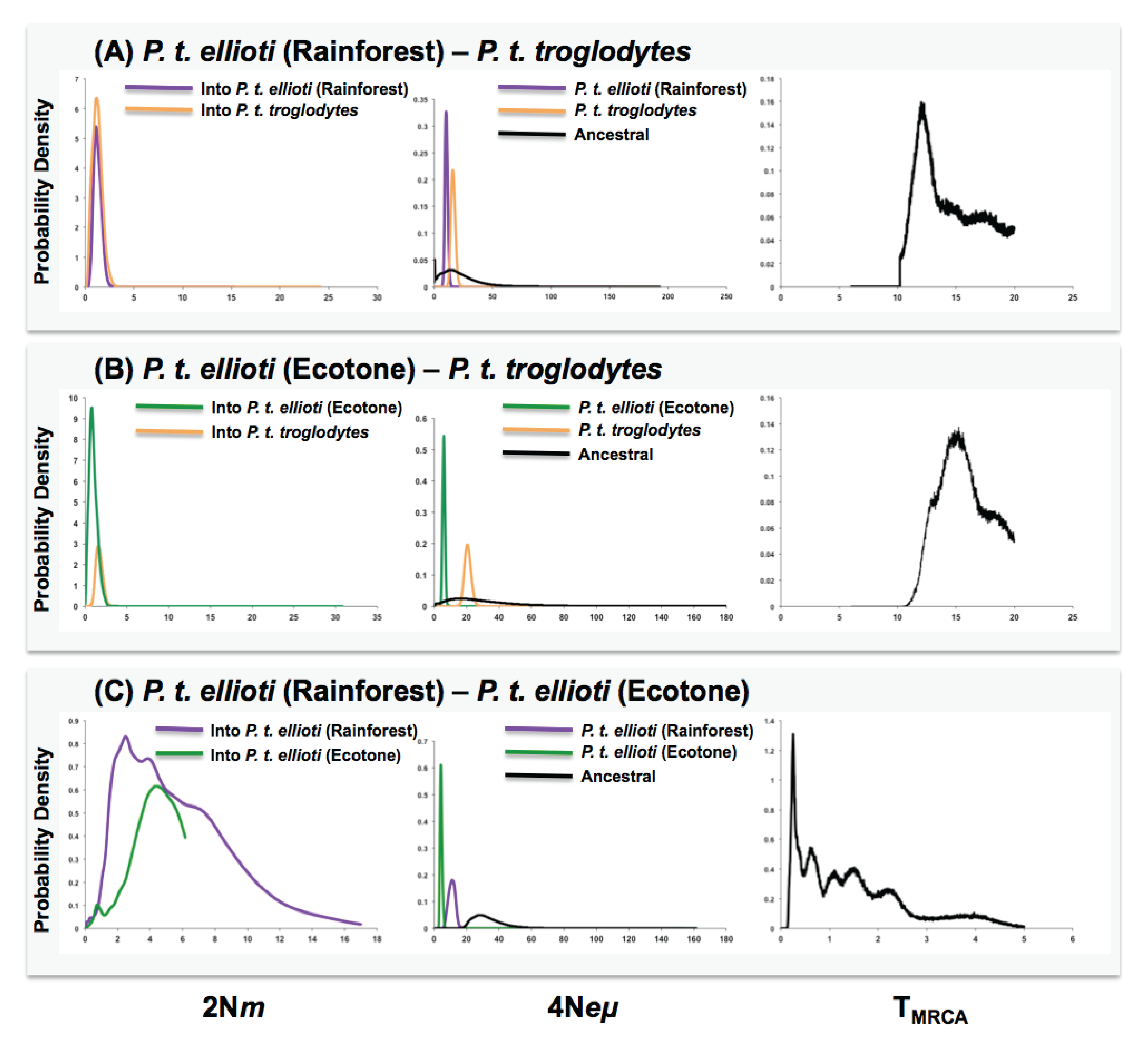

Supplement: Additional file 13: — IMa probability distributions. Posterior probability distributions for three parameters: (i) migration (2Nm); (ii) effective population size (4Neμ); and time of population divergence (TMRCA). Parameters are not scaled according to a mutation rate or chimpanzee generation time. Parameters were tested for three population comparisons: (A) P. t. ellioti (Rainforest) – P. t. troglodytes, (B) P. t. ellioti (Ecotone) – P. t. troglodytes, and (C) P. t. ellioti (Rainforest) – P. t. ellioti (Ecotone). [file 12862_2014_276_MOESM13_ESM.png]
