# Supplementary material for: The population genetics of wild chimpanzees in Cameroon and Nigeria suggests a positive role for selection in the evolution of chimpanzee subspecies
Source: BMC Evol Biol. 2015 Jan 21;15:3. doi: 10.1186/s12862-014-0276-y (PMC4314757; doi:10.1186/s12862-014-0276-y)
Supplement: Additional file 5: — Spatial Analysis of Molecular Variance (SAMOVA) for mtDNA HVRI. [file 12862_2014_276_MOESM5_ESM.docx]

| **Partition** | **Fixation indices** | **Variance Components** | **Percentage of**  **Variation** | **Significance** |
| --- | --- | --- | --- | --- |
|  | North (*P. t. ellioti*) versus south of Sanaga (*P. t. troglodytes*) | | | |
| Among groups (ϕ_CT_) | 00.49 | 08.68 | 49.37 | *p* < 0.05 |
| Among populations in groups (ϕ_SC_) | 00.10 | 00.89 | 05.07 | *p* < 0.05 |
| Within populations (ϕ_ST_) | 00.54 | 08.01 | 45.56 | *p* < 0.05 |
|  |  | | | |
