# Supplementary material for: The population genetics of wild chimpanzees in Cameroon and Nigeria suggests a positive role for selection in the evolution of chimpanzee subspecies
Source: BMC Evol Biol. 2015 Jan 21;15:3. doi: 10.1186/s12862-014-0276-y (PMC4314757; doi:10.1186/s12862-014-0276-y)
Supplement: Additional file 9: — Genetic differentiation ( D 2 ,R ST and δμ 2 ) and migration (2N m ) between populations from across the study area from 21 autosomal microsatellite loci. aMeasures of differentiation were computed between populations as inferred by the cluster analyses. bValues above the diagonal are based on Reynold’s Coancestry Coefficient, D 2, which does not assume that mutations follow the SMM [34]. cValues in underlined italics are migrants exchanged between populations each generation (2Nm). dValues below the diagonal are based on an R ST [35] model of evolution that assumes that mutations follow the SMM. eValues in parentheses are based on δμ 2 model of evolution that also assumes that mutations follow the SMM [36]. fAll population pairwise values were significantly different from null expectations from 10,000 permutations of the data in Alrequin [37]. [file 12862_2014_276_MOESM9_ESM.docx]

|  | Population^abcdef^ | | |
| --- | --- | --- | --- |
| Population | ***P. t. ellioti* (Rainforest)** | ***P. t. ellioti* (Ecotone)** | ***P. t. troglodytes*** |
| *P. t. ellioti* (Rainforest) |  | 0.04 *11.29* | 0.13 *1.28* |
| *P. t. ellioti* (Ecotone) | 0.04 (1.24) |  | 0.08 *2.02* |
| *P. t. troglodytes* | 0.28 (2.67) | 0.20 (2.88) |  |
