# Supplementary material for: The population genetics of wild chimpanzees in Cameroon and Nigeria suggests a positive role for selection in the evolution of chimpanzee subspecies
Source: BMC Evol Biol. 2015 Jan 21;15:3. doi: 10.1186/s12862-014-0276-y (PMC4314757; doi:10.1186/s12862-014-0276-y)
Supplement: Additional file 11: — Summary of results from IMa analysis assuming slow, medium, and fast mutations rates for microsatellite loci. *Demographic parameters were scaled assuming a mtDNA mutation rate of 1.64 x 10^−7^ [89]. There is a great deal of uncertainty in microsatellite mutation rates. Thus, we scaled demographic parameter estimates using slow, intermediate and fast microsatellite mutation rates. Green cell show a demographic estimates calculated using a slow rate of 3.53 x 10^−5^ from Becquet et al. [64]. Blue cells show demographic estimates using an intermediate rate of 7.75 x 10^−5^ calculated from the geometric mean of rates from Wegmann and Excoffier [87]. Red cells show demographic estimates scaled with the fastest mutation rate of 1.6 x 10^−4^ [36,39]. Demographic parameters were scaled assuming a generation time of 20 years for chimpanzees [90]. [file 12862_2014_276_MOESM11_ESM.docx]

| Comparison |  | Migration* | | Population Size* | | | Population Divergence* |
| --- | --- | --- | --- | --- | --- | --- | --- |
|  |  | **Into Pop. 1** | **Into Pop. 2** | **N*e*1** | **N*e*2** | **N*a*** | **T_MRCA_ (ya)** |
| *P. t. ellioti* (Rainforest)  x  *P. t. troglodytes* | *MLE* | 1.10 | 1.15 | 4,521 | 7,107 | 128 | 424,465 |
|  | *95Low* | 0.52 | 0.32 | 3,692 | 5,745 | 468 | 380,490 |
|  | *95High* | 2.37 | 2.53 | 5,694 | 8,639 | 32,301 | 705,606 |
|  | *MLE* |  |  | 2,140 | 3,365 | 60 | 200,979 |
|  | *95Low* |  |  | 1,748 | 2,720 | 222 | 180,157 |
|  | *95High* |  |  | 2,696 | 4,091 | 15,294 | 334,096 |
|  | *MLE* |  |  | 1,071 | 1,684 | 30 | 100,560 |
|  | *95Low* |  |  | 875 | 2,720 | 10 | 90,142 |
|  | *95High* |  |  | 1,349 | 2,047 | 7,652 | 167,166 |
| *P. t. ellioti* (Ecotone)  x  *P. t. troglodytes* | *MLE* | 1.58 | 0.79 | 2,633 | 9,084 | 7,208 | 537,613 |
|  | *95Low* | 0.77 | 0.17 | 1,984 | 7,549 | 1,493 | 426,935 |
|  | *95High* | 2.92 | 2.07 | 3,282 | 10,961 | 45,421 | 694,242 |
|  | *MLE* |  |  | 1,247 | 4,301 | 3,413 | 254,553 |
|  | *95Low* |  |  | 939 | 3,574 | 707 | 202,149 |
|  | *95High* |  |  | 1,554 | 5,190 | 21,506 | 328,715 |
|  | *MLE* |  |  | 624 | 2,152 | 1,708 | 127,366 |
|  | *95Low* |  |  | 470 | 1,788 | 354 | 101,145 |
|  | *95High* |  |  | 778 | 2,597 | 10,761 | 164,473 |
| *P. t. ellioti* (Rainforest)  x  *P. t. ellioti* (Ecotone) | *MLE* | 2.49 | 4.39 | 5,002 | 1,817 | 12,504 | 8,911 |
|  | *95Low* | 0.62 | 1.84 | 3,096 | 1,389 | 8,870 | 4,853 |
|  | *95High* | 13.73 | 8.84 | 6,499 | 2,601 | 26,326 | 142,847 |
|  | *MLE* |  |  | 2,368 | 860 | 5,920 | 4,219 |
|  | *95Low* |  |  | 1,466 | 658 | 4,200 | 2,298 |
|  | *95High* |  |  | 3,077 | 1,231 | 12,465 | 67,636 |
|  | *MLE* |  |  | 1,185 | 430 | 2,962 | 2,111 |
|  | *95Low* |  |  | 734 | 329 | 2,101 | 1,150 |
|  | *95High* |  |  | 1,540 | 616 | 6,237 | 33,842 |
