# Supplementary material for: The population genetics of wild chimpanzees in Cameroon and Nigeria suggests a positive role for selection in the evolution of chimpanzee subspecies
Source: BMC Evol Biol. 2015 Jan 21;15:3. doi: 10.1186/s12862-014-0276-y (PMC4314757; doi:10.1186/s12862-014-0276-y)
Supplement: Additional file 12: — Tests of nested models from IMa for three populations of chimpanzees. aDescription of model tested. bLLR test statistics are calculated as twice the difference in log-likelihood between the indicated model and the full model (Θ1 Θ2 ΘA m1 m2), with degrees of freedom equal to the difference in the number of parameters free to be estimated from the data. c P values are obtained by comparison to the Chi-square distribution. dNumber of free parameters, or degrees of freedom for each model. eWhen the null model is true and has a parameter fixed at the boundary of the parameter space, the expected distribution is a mixture [38]. fBold numbers indicate models that could not be rejected (p > 0.05) by the 2LLR log-likelihood ratio test. [file 12862_2014_276_MOESM12_ESM.docx]

|  | ***P. t. ellioti* (Rainforest) x** | | | ***P. t. ellioti* (Ecotone) x** | | | ***P. t. ellioti* (Rainforest) x** | | |  |
| --- | --- | --- | --- | --- | --- | --- | --- | --- | --- | --- |
|  | ***P. t. troglodytes*** | | | ***P. t. troglodytes*** | | | ***P. t. ellioti* (Ecotone)** | | |  |
| *Model^a^* | *log(p)* | *2LLR^b^* | *P^c^* | *log(p)* | *2LLR^b^* | *P^c^* | *log(p)* | *2LLR^b^* | *P^c^* | *df^d^* |
| Θ_1_ Θ_2_ Θ_A_ m_1_ m_2_ | 0.5571 |  | -- | -0.4396 |  | -- | -3.2524 |  | -- |  |
| Θ_1_ Θ_2_ Θ_A_ m_1_= m_2_ | **0.288^f^** | **0.5382^f^** |  | -5.2149 | 9.5507 | *p*<0.05 | -5.7997 | 5.0947 | *p*<0.05 | 1 |
| Θ_1_ Θ_2_ Θ_A_ m_1_ 0 m_2_ | -68.807 | 138.728 | *p*<0.05 | -9.1186 | 17.3582 | *p*<0.05 | -30.537 | 54.5686 | *p*<0.05 | 1^e^ |
| Θ_1_ Θ_2_ Θ_A_ 0m_1_ m_2_ | -345.83 | 692.782 | *p*<0.05 | -460.52 | 920.155 | *p*<0.05 | **-3.2461^f^** | **-0.0126^f^** |  | 1 ^e^ |
| Θ_1_ Θ_2_ Θ_A_ 0m_1_ 0m_2_ | -460.52 | 922.148 | *p*<0.05 | -460.52 | 920.155 | *p*<0.05 | -460.52 | 914.529 | *p*<0.05 | 2 ^e^ |
| Θ_1_=Θ_2_ Θ_A_ m_1_ m_2_ | -3.6489 | 8.4119 | *p*<0.05 | -368.19 | 735.51 | *p*<0.05 | -8.1951 | 9.8854 | *p*<0.05 | 1 |
| Θ_1_=Θ_2_=Θ_A_ m_1_ 0m_2_ | -4.804 | 10.7222 | *p*<0.05 | -368.21 | 735.539 | *p*<0.05 | -58.209 | 109.912 | *p*<0.05 | 2 |
| Θ_1_=Θ_2_ Θ_A_ m_1_= m_2_ | -6.5279 | 14.1699 | *p*<0.05 | -387.77 | 774.665 | *p*<0.05 | -38.521 | 70.5376 | *p*<0.05 | 2 |
| Θ_1_=Θ_2_ Θ_A_ 0m_1_ 0m_2_ | -460.52 | 922.148 | *p*<0.05 | -460.52 | 920.155 | *p*<0.05 | -460.52 | 914.529 | *p*<0.05 | 3 ^e^ |
| Θ_1_=Θ_2_=Θ_A_ m_1_= m_2_ | -7.6991 | 16.5123 | *p*<0.05 | -394.64 | 788.404 | *p*<0.05 | -165.37 | 324.227 | *p*<0.05 | 3 |
| Θ_1_=Θ_2_=Θ_A_ 0m_1_ 0m_2_ | -460.52 | 922.148 | *p*<0.05 | -460.52 | 920.155 | *p*<0.05 | -460.52 | 914.529 | *p*<0.05 | 4 ^e^ |
| Θ_2_ Θ_1_=Θ_A_ m_1_ m_2_ | -1.9606 | 5.0353 | *p*<0.05 | **-2.0364^f^** | **3.1936^f^** |  | **-4.9328^f^** | **3.3609^f^** |  | 1 |
| Θ_2_ Θ_1_=Θ_A_ m_1_= m_2_ | **-2.2029^f^** | **5.5199^f^** |  | -9.6068 | 18.3345 | *p*<0.05 | -11.206 | 15.9063 | *p*<0.05 | 2 |
| Θ_2_ Θ_1_=Θ_A_ 0m_1_ 0m_2_ | -460.52 | 922.148 | *p*<0.05 | -460.52 | 920.155 | *p*<0.05 | -460.52 | 914.529 | *p*<0.05 | 3 ^e^ |
| Θ_1_ Θ_2_=Θ_A_ m_1_ m_2_ | -1.9532 | 5.0204 | *p*<0.05 | **-2.0255^f^** | **3.1719^f^** |  | -19.928 | 33.3516 | *p*<0.05 | 1 |
| Θ_1_ Θ_2_=Θ_A_ m_1_= m_2_ | **-2.0383^f^** | **5.1907^f^** |  | -5.5511 | 10.223 | *p*<0.05 | -37.807 | 69.1093 | *p*<0.05 | 2 |
| Θ_1_ Θ_2_=Θ_A_ 0m_1_ 0m_2_ | -460.52 | 922.148 | *p*<0.05 | -460.52 | 920.155 | *p*<0.05 | -460.52 | 914.529 | *p*<0.05 | 3 ^e^ |
