# Supplementary material for: The population genetics of wild chimpanzees in Cameroon and Nigeria suggests a positive role for selection in the evolution of chimpanzee subspecies
Source: BMC Evol Biol. 2015 Jan 21;15:3. doi: 10.1186/s12862-014-0276-y (PMC4314757; doi:10.1186/s12862-014-0276-y)
Supplement: Additional file 14: — Microsatellite loci information. aPolymorphic Information Content estimated in CERVUS version 3.0 [91]. [file 12862_2014_276_MOESM14_ESM.docx]

| No. | Marker name | Chrom-osome | Repeat  Unit  (bp) | No. of  Allel-es | Allele  Size Range | PIC^a^ | Forward Primer (5' - 3')  *Reverse Primer (5'-3')* |
| --- | --- | --- | --- | --- | --- | --- | --- |
| 0 | Amelogenin | X/Y | -- | 2 | 106/112 | 0.375 | NED-ACC TCA TCC TGG GCA CCC TGG TT  *AGG CTT GAG GCC AAC CAT CAG* |
| 1 | G00-228-893  D4S1652 | 4 | 4 | 15 | 109-165 | 0.87 | 6FAM - AAT CCC TGG GTA CAT TAT ATT TG  *CAG ACA TTC TTT ATT CTT TAC CTC C* |
| 2 | G00-364-834  D7S1809 | 7 | 4 | 12 | 191-239 | 0.859 | 6FAM - AGG CAA GAG CAG TAG CAA GA  *TCC ACT TTA AAT CAG CAG CC* |
| 3 | G00-218-317  D9S303 | 9 | 4 | 16 | 141-225 | 0.853 | NED - CAA CAA AGC AAG ATC CCT TC  *TAG GTA CTT GGA AAC TCT TGG C* |
| 4 | G00-364-803  D11S1984 | 11 | 4 | 12 | 159-207 | 0.743 | PET - GGG TGA CAG AGC AAA ATT CT  *ACA CCT GGA TCT TGG ACT CA* |
| 5 | GATA7G10.415  D13S317 | 13 | 4 | 13 | 160-220 | 0.789 | VIC - ACA GAA GTC TGG GAT GTG GA  *GCC CAA AAA GAC AGA CAG AA* |
| 6 | G00-228-700  D16S539 | 16 | 4 | 8 | 134-162 | 0.700 | 6FAM - GAT CCC AAG CTC TTC CTC TT  *ACG TTT GTG TGT GCA TCT GT* |
| 7 | G00-364-824  D20S470 | 20 | 4x4x  4x2 | 8 | 221-321 | 0.87 | PET - CCT TGG GGG ATA TAG CCT AA  *TGA GTG ACA GAG TGA TAC CAT G* |
| 8 | Mfd3  APOA2 (D1) | 1 | 2 | 12 | 124-146 | 0.822 | VIC - GGT CTG GAA GTA CTG AGA AAA  *GAT TCA CTG CTG TGG ACC CA* |
| 9 | Mfd23  D16S265 | 16 | 2 | 24 | 075-117 | 0.868 | VIC - CCA GAC ATG GCA GTC TCT A  *AGT CCT CTG TGC ACT TTG T* |
| 10 | HumPla2a | 12 | 3 | 13 | 070-104 | 0.69 | 6FAM - GGT TGT AAG CTC CAT GAG GTT AGA  *GTC CTA GGA GCT AGA GAT ACA GC* |
| 11 | GATA43A04  D1S1653 | 1 | 4 | 16 | 103-175 | 0.816 | VIC-GGA AAG CCT GTA GGA AGA GG  *CCT GGA TGA CAG AGT GCT CT* |
| 12 | GATA164B08P  D3S4545 | 3 | 4 | 25 | 182-286 | 0.939 | VIC-CTG TGA TCA CAC CAC TGC AG  *TGG GGT ATC CTG TGT CAG AGC* |
| 13 | ATA28F03  D4S3248 | 4 | 4 | 11 | 232-272 | 0.841 | 6FAM-TTC AGG AGT TTA GCT TTC TAT GC  *CTA CAC CAT CAG TAC TCA CTA GGC* |
| 14 | GATA43C11  D7S1804 | 7 | 4 | 18 | 192-284 | 0.898 | 6FAM-TTC AAG TGG TTG GGT TCA CT  *TGG GTC TAG TCC AGT GGT GT* |
| 15 | GATA14E09  D8S2324 | 8 | 4 | 9 | 175-207 | 0.778 | NED-TGA AAA CAT AGT ACA ATG AAC ATC C  *GTC ATA ATA TCT GCC AAT GAT TG* |
| 16 | TCTA017M  TCTA017M | 9 | 4 | 14 | 150-206 | 0.773 | 6FAM-CAG CAT GAG AGT GGT TGA GG  *ATG GTG TCA AAC ATG AAT TAG GC* |
| 17 | ATA27A06P  D12S1042 | 12 | 3 | 8 | 110-131 | 0.754 | VIC-TAT GAC GGT GCA CCA CAT AC  *AAC CTG CAT GTT CTG CAT AT* |
| 18 | GGAA21G11L  D14S617 | 14 | 4 | 12 | 121-169 | 0.771 | 6FAM-TTT TAG GTG GCC ACC ATC TA  *CCA GTT TAG GCA ACA GAA CA* |
| 19 | GATA50G06  D15S643 | 15 | 4 | 17 | 192-280 | 0.894 | PET-ATA CCT GGA GTC CTT GGT CC  *AAC AGC TTT AAA ACC TCA ATG C* |
| 20 | GATA8C04  D17S974 | 17 | 4 | 7 | 188-212 | 0.718 | PET-AGA CCC TGT CTC AGA TAG ATG G  *TAA AAT AGA AAG TGC CCC TCC* |
| 21 | ATTT030 | 6 | 4 | 8 | 101-129 | 0.677 | 6FAM-GGG TCC ATT AGT TGA GTA TGC AG  *GCC TGG CAG ACA AGA GTG AA* |
